# Record-setting heavy precipitation occurrence in 2024

**DOI:** 10.1126/sciadv.aeb1692

**Published:** 2026-10-09

**Authors:** Chad W. Thackeray, Jesse Norris, Alex Hall, Mark D. Zelinka, Stephen Po-Chedley

**Affiliations:** ^1^ Department of Atmospheric and Oceanic Sciences, University of California, Los Angeles, Los Angeles, CA, USA.; ^2^ Atmospheric, Earth, and Energy Division, Lawrence Livermore National Laboratory, Livermore, CA, USA.

## Abstract

Precipitation extremes have increased globally in recent decades, consistent with a warming climate. We find that 2024 experienced a record-high frequency of heavy (≥99th percentile; *F*_P≥99_) precipitation events globally based on MSWEP (Multi-Source Weighted-Ensemble Precipitation) observations from 1979 to 2025. This extends a long-term trend of ∼0.03%/decade in *F*_P≥99_, with similar but more uncertain increases for the 99.9th percentile. Unlike prior high *F*_P≥99_ years, 2024’s record was more globally distributed and emerged from a distinct seasonal evolution. El Niño and rare concurrent multi-basin tropical ocean warmth drove elevated extremes early in the year, before lagged global tropospheric responses and long-term warming sustained and amplified extremes through boreal summer. Climate models indicate that years with 2024-level *F*_P≥99_ typically require either stronger El Niño forcing or concurrent multi-basin sea surface temperature (SST) anomalies. Diagnostic projections within constrained ensemble members suggest that ∼40% of the 2024 anomaly aligns with global mean temperatures and ∼20% with El Niño variability, with some of the remainder linked to basin-scale SST structure.

## INTRODUCTION

2024 was the hottest year on record globally (*1*, *2*), surpassing the prior year that itself was a record, and continuing a long-term trend of warming since the preindustrial period (*3*). In addition to global surface temperature, 2024 set record-high globally averaged values for sea surface temperature (SST), ocean heat content (over the top 2000 m), sea level, land surface temperature, lake surface temperature, and tropospheric temperature (*4*, *5*). An expected, and impactful, a consequence of this warming is more frequent and intense extreme precipitation events (*6*–*11*). This change largely occurs because warmer air has a greater moisture-holding capacity, ∼7%/K following Clausius-Clapeyron (CC) scaling (*12*–*14*). Although global mean precipitation is constrained by the atmospheric energy budget (where enhanced longwave cooling to space is balanced by latent heating) (*15*, *16*), individual extreme events are less sensitive to these global constraints and thus exhibit increases closer to CC scaling.

It is expected that the rarest precipitation events will experience the largest increases in intensity and frequency (*17*, *18*), with increased extremes due to occur across nearly all latitudes (*19*, *20*). These changes began to become apparent in observations over certain regions roughly two decades ago (*21*, *22*), but it was not until more recently that they became detectable in global averages (*23*–*25*). For example, in Thackeray *et al.* (*25*) (hereafter T22), we noted an increased occurrence of heavy precipitation events (frequency of events greater than the local 99th percentile of daily precipitation from 1980 to 2014; defined as *F*_P≥99_) over recent decades both globally and over land regions with long-term measurements. This metric represents the frequency with which daily precipitation exceeds a local 1-in-100-day threshold derived from the 1980 to 2014 period (we maintain this baseline here for consistency with T22; see Materials and Methods). Because this threshold is defined from a fixed baseline, we expect *F*_P≥99_ to exceed 1% under a warming (nonstationary) climate. Moreover, because the 99th percentile lies in the steeply declining tail of the precipitation distribution, modest shifts in precipitation intensity can produce disproportionately large increases in exceedance frequency. Thus, trends in *F*_P≥99_ reflect both thermodynamic intensification and shifts in the distribution’s shape. From 1980 to 2017, we found that *F*_P≥99_ increased globally at a rate of 0.028%/decade in observations, largely in line with global climate model (GCM) trends (∼0.030 ± 0.008%/decade).

The observed *F*_P≥99_ time series also exhibits notable interannual variability linked to naturally occurring climate patterns, such as the El Niño–Southern Oscillation (ENSO), as evidenced by spikes in the frequency of global precipitation extremes during and in the aftermath of strong El Niño events (*26*). However, because of the rarity of such events in the satellite record (1982–1983, 1997–1998, 2015–2016, and 2023–2024), our understanding of extreme precipitation behavior during strong El Niño events remains somewhat limited. The most recent El Niño, which began in 2023 and persisted into 2024 (*26*, *27*), may therefore offer further insight into how background warming is affecting the response of precipitation extremes to the warm phase of ENSO. This is important because several studies point to greater ENSO variability in the future (*28*–*30*). Recent improvements in the timeliness of precipitation dataset availability allow us to update the analysis of T22 (which ended in 2017) through 2025, comment on the change in trends in observations and models, and investigate the relationship between ENSO and *F*_P≥99_ in multiple GCM large ensembles (LENSs).

## RESULTS

### Historical precipitation trends

We begin by examining global trends in the frequency of heavy precipitation events (*F*_P≥99_), extending the analysis of T22 through 2025. Observations primarily come from an updated version of the Multi-Source Weighted-Ensemble Precipitation (MSWEP) dataset (*31*) (see Materials and Methods). The extended time series reveals a robust upward trend alongside large interannual variability, with the most prominent historical peaks preceding 2024 coinciding with the major El Niño events of 1983 and 1998 (Fig. 1, A and C). In 2024, the confluence of long-term warming, a strong El Niño, and multi-basin ocean warmth helped produce a record global *F*_P≥99_ value of 1.2%, representing a 20% increase relative to the 1980 to 2014 baseline. Further analysis of this year is presented in a later section. Over the full 1979 to 2025 period, the observed trend is 0.031%/decade, slightly higher than the previously reported 1980 to 2017 trend. This observed trend is consistent with the ensemble mean from a suite of LENSs. Ensemble-mean simulated trends range from 0.022 (E3SM2) to 0.038%/decade (CanESM5) across models (Fig. 1B), although the influence of internal variability introduces substantial spread in individual realizations.

**Fig. 1.**
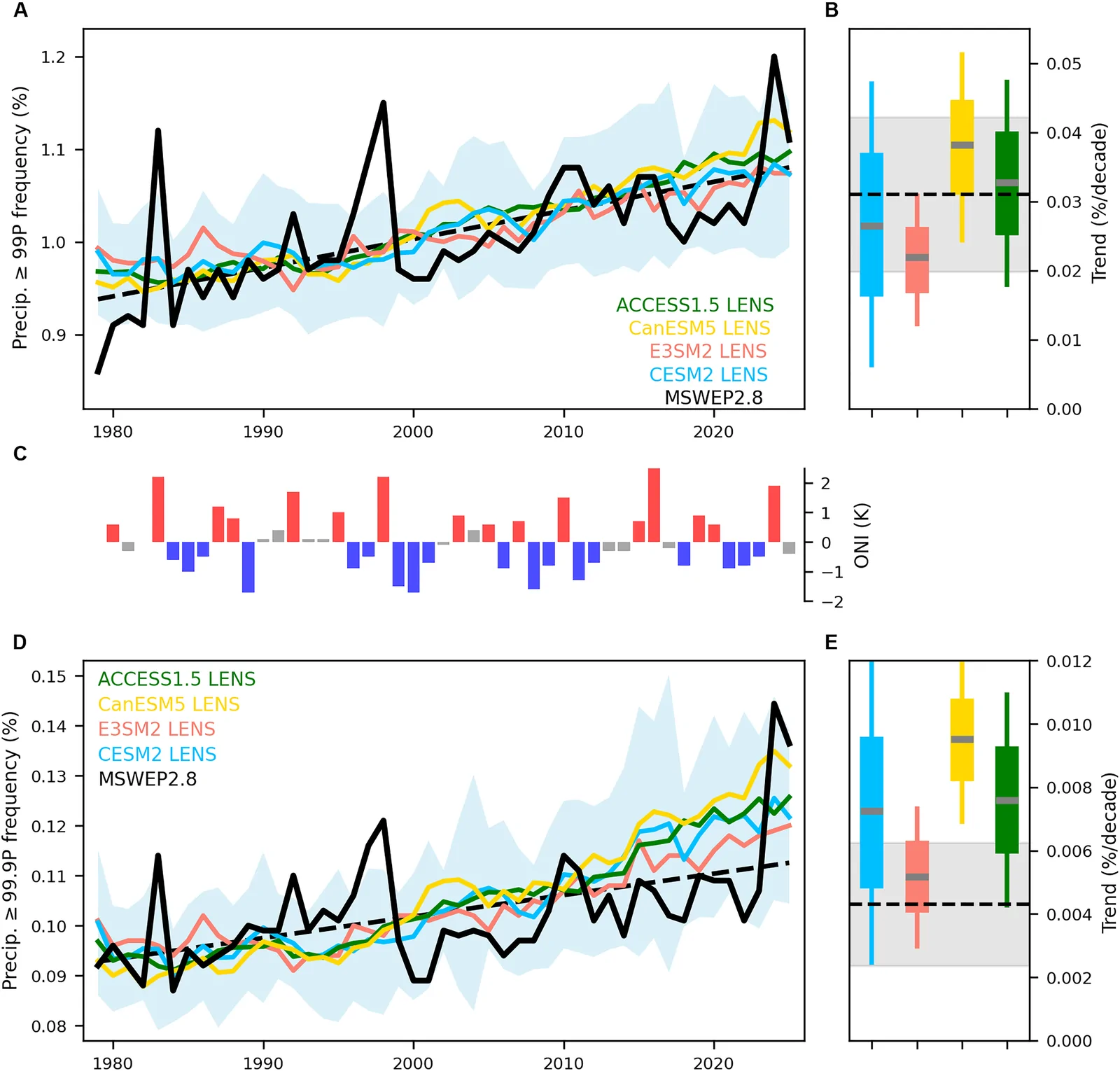
Historical time series of global heavy precipitation frequency from observations and models. Comparison of the observed (black line) and simulated (colored lines) frequency of precipitation events greater than or equal to the (**A**) 99th percentile and (**D**) 99.9th percentile. A linear trend line is shown for observations with a dashed black line. Light blue shading represents ±1 SD from the ensemble mean for one of the LENSs (CESM2). Observations are derived from MSWEP, version 2.8. (**B**) Distribution of historical trends in *F*_P≥99_ for each dataset are shown by colored bars, where the wider portion encapsulates ±1 SD from the mean (gray line) whereas the narrow portion shows ±2 SDs. The horizontal dashed line shows the observed trend, whereas gray shading represents the regression slope uncertainty (95% confidence interval). (**E**) Same as (B), but for *F*_P≥99.9_. (**C**) Bar chart of the DJF-mean ONI where each bar is color coded based on El Niño, La Niña, or neutral conditions.

Given that the rate of change is expected to be larger at higher percentiles (*18*), we also calculate trends in the frequency of extreme precipitation events (greater than the 99.9th percentile; *F*_P≥99.9_) (Fig. 1D). For this metric, the 2024 anomaly is especially pronounced, far exceeding previous highs. The observed *F*_P≥99.9_ value in 2024 reached 0.144%, a 44% increase relative to climatology. This metric represents the frequency of events that occur roughly once every 1000 days and is therefore zero in most locations in a given year. Owing to the limited number of events that inform the 99.9th percentile over the observational record, estimates of *F*_P≥99.9_ trends are more sensitive to the choice of baseline period. Within this uncertainty, long-term observed trends are smaller than those simulated by the LENS models, which average 0.0074%/decade and have ensemble means spanning 0.0052 to 0.0097%/decade (Fig. 1E), whereas observed estimates based on the 1980 to 2014 baseline yield a trend of 0.0043%/decade. Although lower than the ensemble means, the observed trend falls within the natural variability envelope spanned by individual members in three of the four ensembles. As a result, comparisons of *F*_P≥99.9_ trends between observations and models should be interpreted with caution given uncertainties in observational sampling, potential residual biases in precipitation datasets, the confounding effects of internal variability on single realizations, and possible nonlinearities in model precipitation responses to warming (*32*).

We next assess trends over land, where precipitation extremes carry heightened societal relevance. In addition to MSWEP, we incorporate two long-term land-only observational datasets (CPC and CHIRPS) over their common period of reliable agreement (1983 to 2025; see Materials and Methods). This is important because inter-dataset uncertainty can be large for some precipitation metrics (*33*, *34*). On the basis of the multi-dataset mean, *F*_P≥99_ reached a record-high over land in 2024, and the long-term trend over this period is 0.018 ± 0.006%/decade (fig. S1). As in T22, the MSWEP trend lies near the center of the observational range. In contrast, the simulated land-area trends from the LENS models are higher than both the observed trends and their global counterparts (LENS mean: 0.044 ± 0.009%/decade). The range of land trends produced by each LENS is also smaller than for the global domain, illustrating a weaker influence of internal variability on terrestrial *F*_P≥99_ compared to the global average, which is heavily influenced by the highly variable tropical Pacific. Similar results are found for *F*_P≥99.9_, although observational uncertainty is larger in this case.

The spatial distribution of changes reveals more nuanced patterns than a simple land-ocean contrast, but caution should be taken when interpreting change at individual locations as substantial observational uncertainty exists at these scales. A prominent feature is a band of strongly increasing *F*_P≥99_ across much of the intertropical convergence zone (ITCZ), accompanied by large reductions south of the equator, particularly over the eastern tropical Pacific (Fig. 2A). Previous studies have documented similar patterns of change for mean precipitation (*35*), including an intensification of ITCZ rainfall (*36*). At higher latitudes, a coherent wetting signal is evident across the Arctic (60° to 90°N), consistent with sea ice retreat and the increasing atmospheric moisture capacity. When zonally averaged, ∆*F*_P≥99_ has increased across most latitudes, except in the Southern Hemisphere (SH) tropics and a narrow band approaching 30°N (Fig. 2B). The largest overall increase occurs just north of the equator, whereas extratropical trends generally intensify moving poleward from the tropics. The simulated trends in *F*_P≥99_ generally resemble observations, but it should be noted that there is considerable uncertainty amongst observational datasets across land regions (fig. S2). There is, however, typically good inter-model agreement on the sign of zonal changes.

**Fig. 2.**
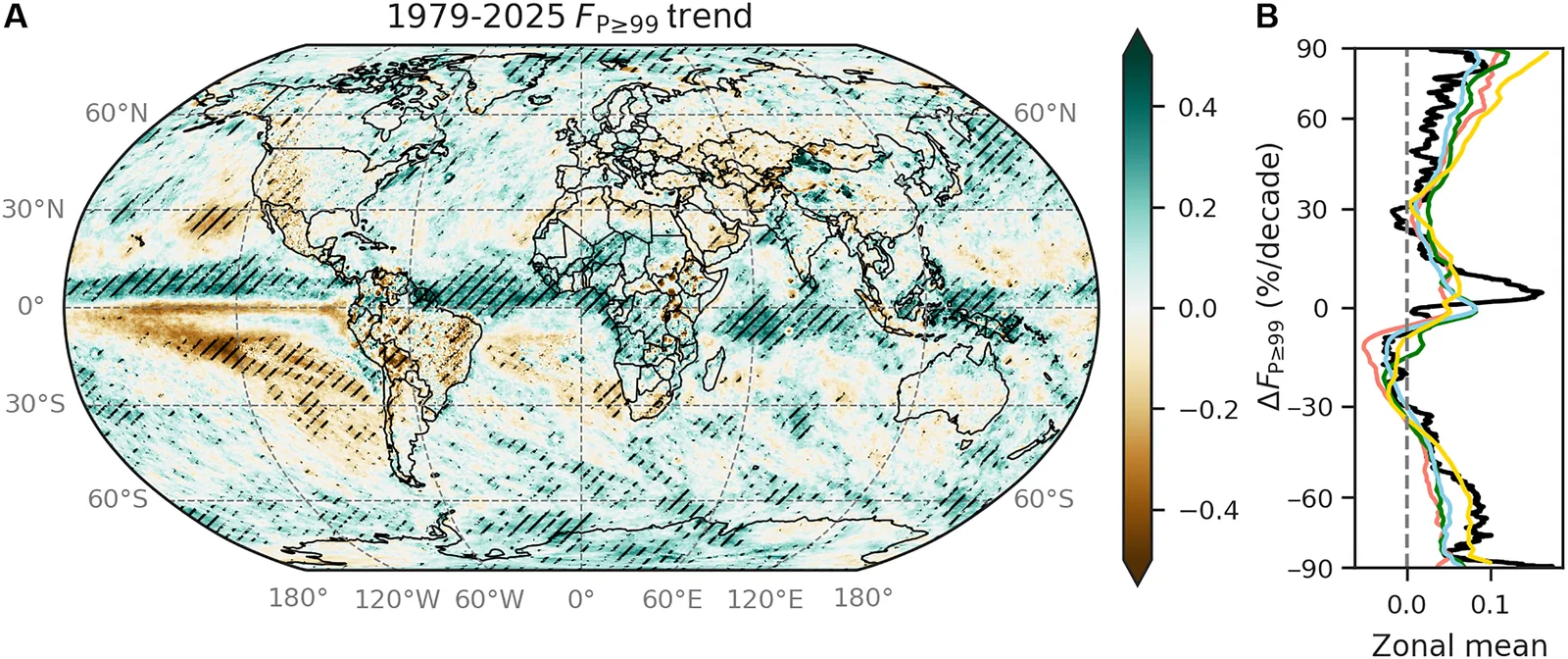
Observed and simulated spatial trends in *F*_P≥99_. (**A**) Global map of observed *F*_P≥99_ trends from MSWEP. Black hatching overlays areas where the trend is significant (*P* < 0.05). The hatching density is decreased for visualization purposes. (**B**) Zonal mean trends in *F*_P≥99_ according to observations (black line) and a collection of GCM LENS means (colored lines as in Fig. 1).

### Extreme precipitation in 2024

Next, we examine the conditions that contributed to record-setting heavy and extreme precipitation occurrences in 2024. We characterize this extremity using two complementary metrics: local annual exceedance counts and global monthly frequencies [(*F*_P≥*n*_); see Materials and Methods]. The year began with strong El Niño conditions, which had developed in the latter half of 2023 and peaked during November to January with a +2.1K Oceanic Niño Index (ONI) (*26*). This marked the first El Niño in several years, following three straight La Niña years (Fig. 1C). A transition to ENSO-neutral conditions occurred by May to July, with a brief dip into weak La Niña territory at the end of the year. Against this El Niño backdrop, the frequency of heavy precipitation days was markedly elevated around the globe. Roughly 1% of days are expected to exceed the 99th percentile threshold in any given location during 1980 to 2014 (∼3.65 days/year), and thus theoretically 49% of locations would exceed this threshold four or more times in a baseline year, according to MSWEP, 2024 saw 57% of the globe experience at least 4 such days. This represents a substantial departure from the theoretical binomial expectation for local annual exceedance counts, which averages 47% over the 1980 to 2014 period due to a nonstationary baseline climate (fig. S3). The area experiencing double the expected frequency (≥7.3 days) also more than doubled relative to the 1980 to 2014 average (13.5% versus 6.3%). A similar expansion was seen in days exceeding the 99.9th percentile, with 36% of the globe experiencing at least one such event, up from a theoretical expectation of 31% (28% empirical baseline average).

Despite the waning El Niño, anomalously high heavy precipitation occurrence persisted through most of the year. Climatologically, the historical baseline of *F*_P≥99_ is largest in boreal summer (July to August; fig. S4A) when global temperatures and atmospheric moisture capacity peak. Because global tropospheric temperatures and column water vapor systematically lag boreal winter equatorial Pacific SSTs by 3 to 6 months (*37*–*39*), the El Niño effectively primes the global atmosphere, sustaining an elevated *F*_P≥99_ through the first half of 2024. A month-by-month linear trend analysis further reveals a stark seasonal asymmetry: Although January to April extremes are heavily modulated by internal variability and show no significant long-term trends, August to October exhibit significant positive trends (fig. S4C). This seasonal partitioning in the drivers of global *F*_P≥99_ explains why 2024’s anomalies persisted throughout most of the year, whereas other strong El Niño years like 1983 and 1998 saw *F*_P≥99_ collapse during boreal summer (fig. S4A). Fueled by this combination of a lagged ENSO baseline and a strong anthropogenic warming trend, monthly *F*_P≥99_ exceeded 1.2% in seven separate months, peaking at 1.5% in August and subsequently returning to roughly normal levels by the end of the year. Similarly, *F*_P≥99.9_ also peaked in August (Fig. 3B), highlighting the increased occurrence of both heavy and extreme precipitation events. Given that *F*_P≥99.9_ in 2024 surpassed the prior record by a substantial margin (Fig. 1), we next look at the spatial distribution of extreme events during this year.

**Fig. 3.**
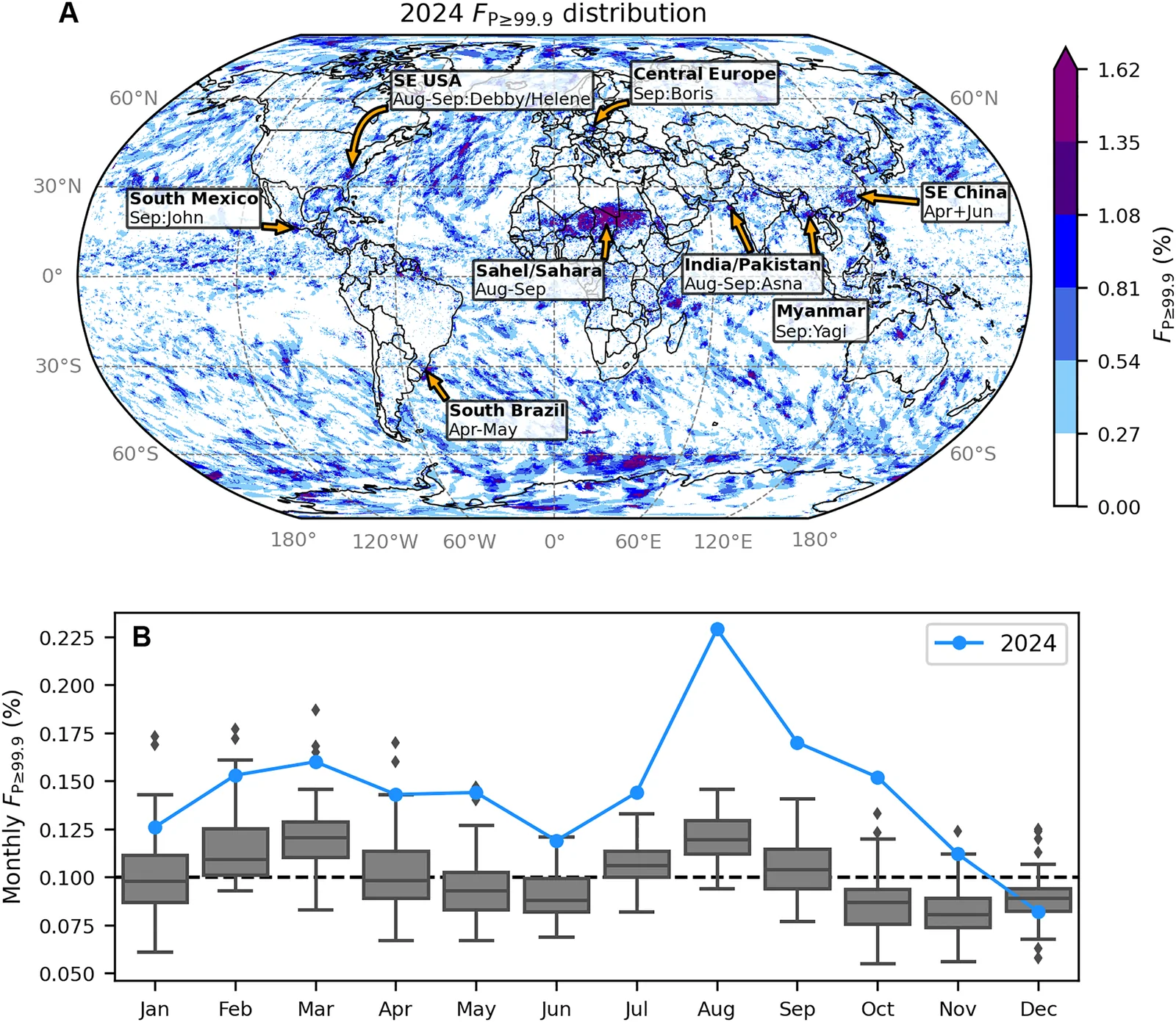
Distribution of extreme precipitation events in 2024. (**A**) Annotated map of observed *F*_P≥99.9_ from 2024. For reference, if 1 day during the year was greater than or equal to the 99.9th percentile, then the displayed value would be 0.27%. Labels point out the timing and location of particularly anomalous or impactful events with the storm name if one was given. (**B**) Boxplot of monthly global mean *F*_P≥99.9_ from MSWEP over 1979 to 2024. Values for 2024 are illustrated by the blue line.

We find that an unusually large number of *F*_P≥99.9_ events occurred in the Sahel-Sahara transition zone, where expansive areas experienced persistent extreme rainfall during August to September (Fig. 3A and fig. S5). Although extreme statistics in such arid regions are subject to higher uncertainty due to rainfall intermittency and sparse observational coverage, notably, even when this area is excluded from global averaging, we still see record statistics for 2024. There were also more localized extreme precipitation events, which drove major flooding in southeastern China (*40*), Central Europe, and southern Brazil (*41*). The effects of slow moving or stalling tropical cyclones were evident as well, with notable anomalies during Aug-Sep around Myanmar (Typhoon Yagi), the India-Pakistan border (Cyclone Asna), southern Mexico (Hurricane John), and the southeastern US (Hurricanes Debby and Helene). Increased heavy precipitation during 2024 is also reflected in magnitude-based metrics, such as the 99th percentile of daily precipitation (fig. S6). For instance, the global mean 99th percentile of 27.81 mm/day exceeds the value of 25.73 mm/day for the climatological period.

### Influence of tropical SST structure

From Fig. 1, it is evident that ENSO has a strong influence on *F*_P≥99_. To better understand how tropical SSTs affect the spatial distribution of *F*_P≥99_, we contrast 2024 with the prior standout cases of 1983 and 1998 (*42*–*44*). Both 1983 and 1998 feature *F*_P≥99_ maps that are dominated by anomalously high values (>10%) in the eastern Pacific (EP) (fig. S7). This enhancement occurs because strong EP warming shifts deep convection toward the equator (*45*, *46*), where climatological *F*_P≥99_ thresholds are lower. A similar picture emerges for *F*_P≥99.9_. These events reflect the canonical EP El Niño regime, characterized by strong Niño 1+2 warming and a weakened zonal SST gradient.

The 2016 El Niño illustrates that ONI magnitude alone does not determine the *F*_P≥99_ response. Despite 2016’s record-high ONI, its *F*_P≥99_ pattern differs drastically from that of 1983 and 1998. The 2016 event featured a greater westward extension of anomalous SSTs than these prior years (*47*), which were strongest in the Niño 1+2 and 3 domains (*48*). Consequently, the 2016 *F*_P≥99_ pattern more closely resembles that of central-Pacific El Niño years, such as 1992 and 2010 (fig. S8). *F*_P≥99_ differences among El Niño events therefore depend on SST structure and basin-wide context.

Similarly, we find that 2024 also lacks a pronounced EP signature (fig. S9). Relative to 1983 and 1998, 2024 exhibits higher annual mean sea level pressure (SLP) (up to 150 Pa) and cooler SSTs (up to 3°C) off the coast of South America (fig. S9, C and D), which weaken the east-west SST gradient and alter the Walker circulation. A correlation analysis between *F*_P≥99_ and the Niño 1+2 minus Niño 4 SST gradient (fig. S10) confirms that EP *F*_P≥99_ anomalies occur during strongly positive gradients. Whereas 1983 and 1998 align with this relationship, 2024 represents a departure from this canonical Pacific-centric forcing. This structural discrepancy arises because the annual mean obscures the intense December-January-February (DJF) peak by blending it with the subsequent cooling phase. Ultimately, the preceding boreal winter governs the global atmospheric priming rather than the annual mean state.

To explain the broader 2024 *F*_P≥99_ response, we quantify detrended SST anomalies across major tropical basins (Fig. 4). We find that 2024 combined El Niño conditions with simultaneous positive anomalies across the tropical Atlantic and Indian Oceans (20°S to 20°N). Since 1979, only 1998 shows comparable basin-mean anomalies exceeding 0.5K across all three basins during DJF. However, 2024 also featured vast extratropical warmth; the global mean DJF SST anomaly reached 0.32K, substantially exceeding prior El Niño peaks in 1983 (0.22K), 1998 (0.19K), and 2016 (0.17K). We suggest this pan-tropical warming enhanced atmospheric moisture and large-scale ascent beyond the canonical ENSO response, promoting a more globally distributed increase in *F*_P≥99_ that was sustained into the summer by the sub-yearly persistence of extra-Pacific warmth (fig. S4). Consistent with this, 2024 exhibits elevated zonal mean heavy and extreme precipitation across nearly the whole Northern Hemisphere (NH) and the SH extratropics (fig. S9), a spatial distribution that aligns with the late-boreal-summer peak of the long-term secular trend in the NH extratropics. This difference is most pronounced over polar regions, which we expect to experience enhanced rainfall in a warming climate due to a combination of local and remote effects (*49*, *50*).

**Fig. 4.**
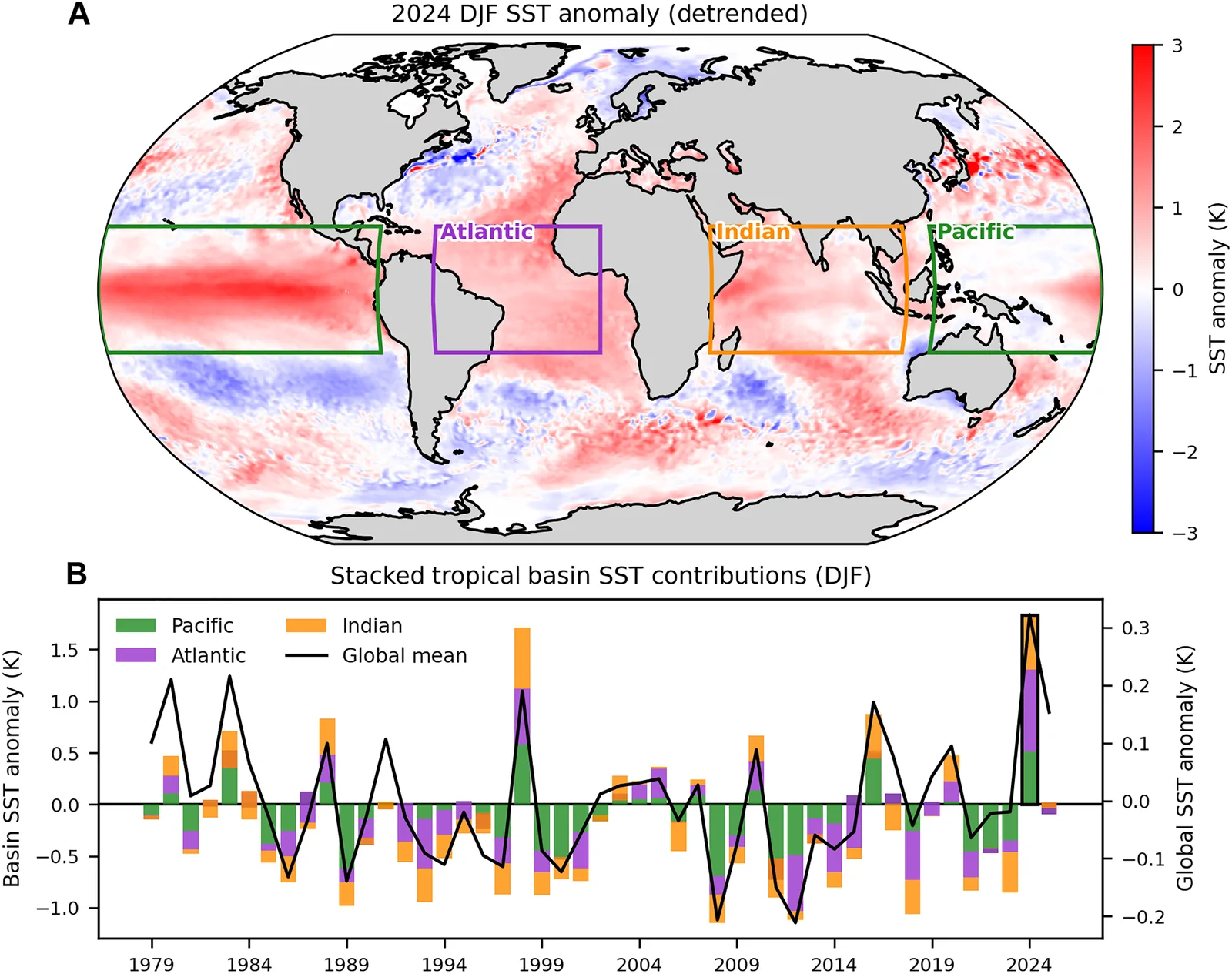
Distribution of anomalous DJF mean SSTs in 2024. (**A**) Map of 2024 DJF SST anomalies (detrended) from ERA5. Colored boxes roughly denote major tropical (20°S to 20°N) basins for the Pacific, Atlantic, and Indian Oceans. (**B**) Stacked bar chart of historical tropical SST anomalies for the three basins from 1979 to 2025. Darker or blended shading (e.g., darker orange or purple) occurs during years with opposing positive and negative anomalies across the basins due to the stacked bar structure. The black line denotes the global mean detrended SST anomaly to help illustrate extratropical SST changes.

Given the limited number of strong El Niños over the satellite record, we use historical data from the GCM LENSs to draw further insights on the rarity of 2024 and the conditions required to produce such extremes. To provide a direct temporal baseline for the observed event, the collective, multimodel LENS provides 135 *F*_P≥99_ values for 2024 ranging from 0.95 to 1.54%. Although this calendar-year comparison inherently combines internal variability with model trend uncertainty, it evaluates the range of potential conditions within the models’ contemporary transient climate states. The lower bound illustrates that even simulated strong La Niñas now resemble average years from earlier decades because of background warming. In this simulated distribution, the observed 2024 *F*_P≥99_ (1.20%) corresponds to the 91.4th percentile.

We find that models can reproduce the observed 2024 *F*_P≥99_ value earlier in the time series, but this typically requires a much stronger El Niño than observed (Fig. 5). Only 125 of the 10,125 simulated years (from 1950 to 2024) exhibit *F*_P≥99_ ≥ 1.2%, and most of these cases (102/125) have a DJF ONI greater than the observed 1.9K. The median DJF ONI among these years is 3.0K (mean: 2.8 ± 1.0K). However, these events generally occur at lower global mean surface temperature (GMST) anomalies than the 0.84K observed in 2024 (relative to 1980 to 2014). For example, in ACCESS-ESM1.5, high *F*_P≥99_ years occur with an average GMST anomaly of 0.67K, whereas in CESM2, they occur with an average of 0.40K due to its stronger ENSO variability. These results indicate that, although a stronger El Niño is generally required to produce 2024-level extremes in a cooler background climate, a warmer background state reduces the ENSO amplitude needed to achieve comparable *F*_P≥99_ values.

**Fig. 5.**
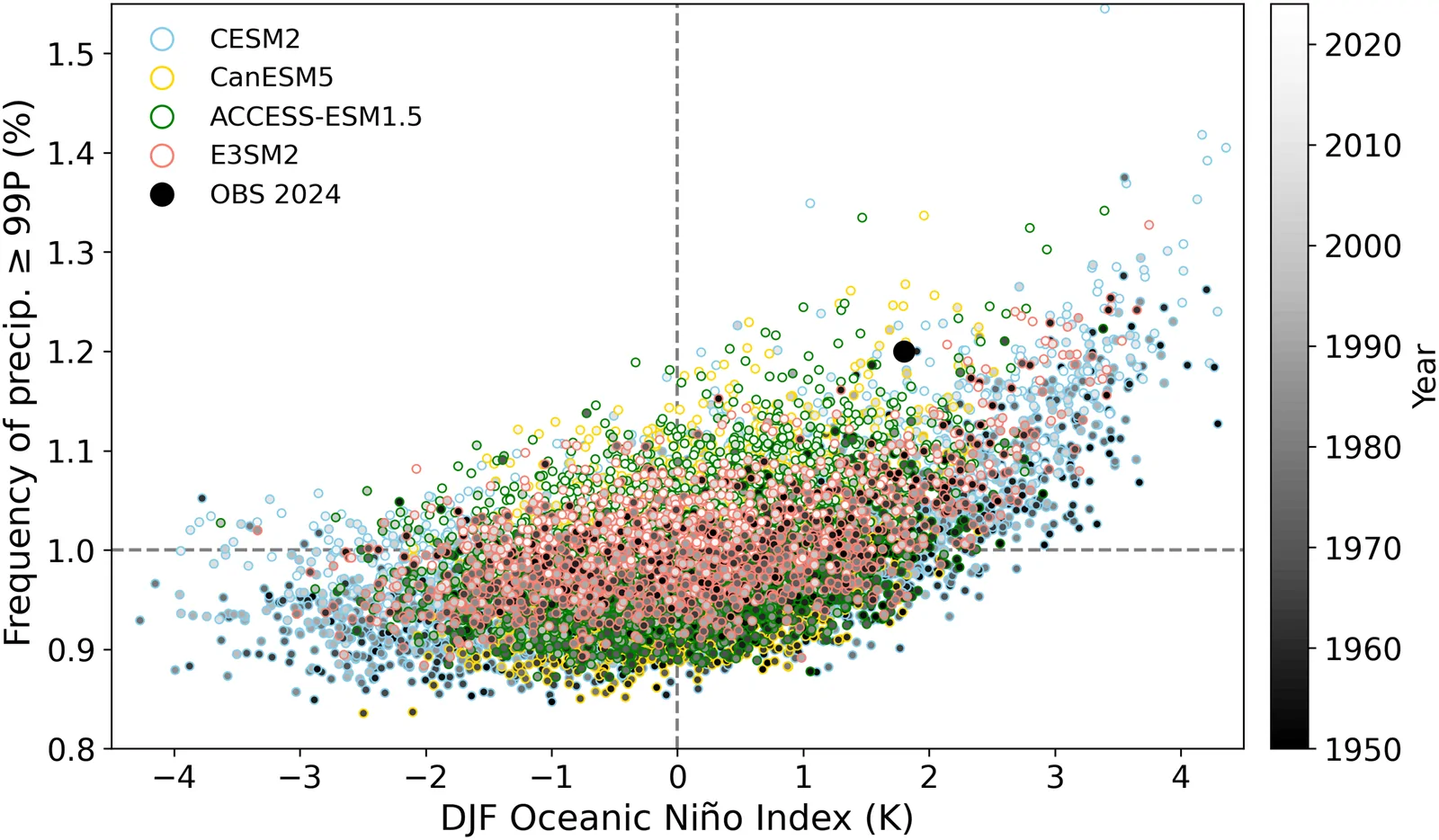
Impact of ENSO on heavy precipitation occurrence in GCM LENSs. Scatterplot of the relationship between the DJF ONI and the frequency of precipitation ≥ 99th percentile from a collection of GCM LENSs for the period of 1950 to 2024. Each dot represents a different year from a different simulation, whereas the points are color coded by their year. The black dot situates observations from 2024 within the model variability space.

The SST configurations associated with the 125 high *F*_P≥99_ years further clarify this modulation. We find that nearly all of these years exhibit positive DJF SST anomalies across the eastern equatorial Pacific, whereas more than half feature positive anomalies across the Atlantic and Indian Oceans (fig. S11), resembling the multi-basin structure observed in 2024 (Fig. 4A). Within the two LENSs that best capture the observed *F*_P≥99_ trend, many of the largest *F*_P≥99_ years satisfy concurrent thresholds for warm anomalies approximating 2024 conditions (Fig. 6). This indicates that GCMs can generate 2024-like extremes through similar multi-basin warming rather than Pacific forcing alone. CESM2 produces these analogs more frequently than ACCESS-ESM1.5, consistent with its stronger ENSO variability. Several of the other most anomalous high *F*_P≥99_ years correspond to El Niño events that intensify throughout the year, transitioning from moderate DJF anomalies (>0.5K) to very strong conditions (>2.0K) by the following DJF. Collectively, the LENS results show that 2024 occupies a regime in which moderate-to-strong El Niño conditions combine with elevated background warming and multi-basin SST anomalies to produce exceptional precipitation extremes.

**Fig. 6.**
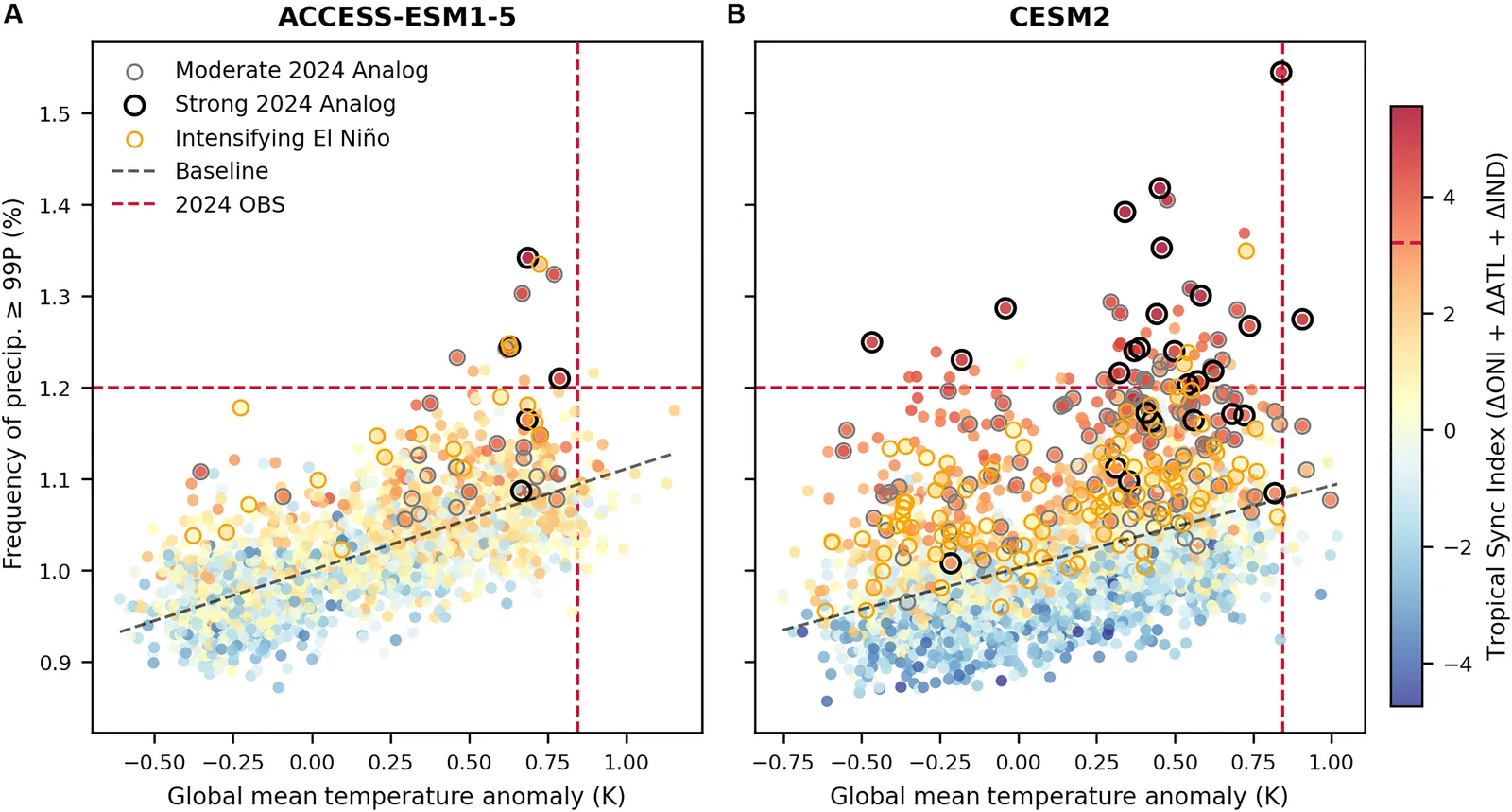
Historical relationship between global mean temperatures and *F*_P≥99_. Scatterplots of annual GMST anomalies (relative to a baseline climate of 1980 to 2014) and annual global mean *F*_P≥99_ from all simulated years during 1979 to 2024 for (**A**) ACCESS-ESM1.5 and (**B**) CESM2. Each point is color coded by an aggregate measure of detrended tropical SST anomalies (called the Tropical Sync Index), which we define as the sum of the DJF anomalies for the ONI, the Atlantic tropical basin (ATL), and the IND. Years with either strongly or moderately analogous conditions to 2024 are outlined in black and gray, respectively. Strong analogs are defined as having DJF ONI > 1.5K, DJF ATL > 0.4K, and DJF IND > 0.4K, whereas moderate analogs have lower thresholds of 0.2K for the ATL and IND basins. Years with an intensifying El Niño, defined as transitions from moderate DJF ONI (>0.5K) to very strong ONI (>2.0K) by the following DJF, are also highlighted (orange outline). The black dashed line illustrates the linear line of best fit for relationships, whereas observed values for 2024 are shown with red dashed lines (with an observed Tropical Sync Index value of 3.2K).

### Projection of the 2024 *F*_P≥99_ anomaly onto warming and ENSO variability

Last, using data from the two GCMs that best mirror the observed *F*_P≥99_ trend, and only those ensemble members whose historical *F*_P≥99_ trends are consistent with observations, we examine how the 2024 *F*_P≥99_ anomaly projects onto global temperatures versus ENSO variability. We emphasize that this regression framework is intended as a diagnostic projection of variability within constrained ensemble members, rather than a detection and attribution analysis. Using a multiple linear regression model with GMST and DJF ONI as predictors (fit separately to individual ensemble members; see Materials and Methods) indicates that ∼39 to 42% of the anomalous 2024 *F*_P≥99_ projects onto the GMST term (Table 1). The ENSO-projected fraction is more GCM dependent, ranging from 17 ± 7% in ACCESS-ESM1.5 (*n* = 21) to 27 ± 6% in CESM2 (*n* = 15). Although this simple model captures much of the variability in simulated *F*_P≥99_ (*R*^2^ = 0.56 to 0.62), it underestimates in its prediction for *F*_P≥99_ in 2024 when given the observed GMST and ONI anomalies by ∼0.07%.

**Table 1.** Diagnostic estimation of key metrics statistical relevance to changes in *F*_P≥99_. Summary of results from the linear diagnostic framework for each of the two OLS model designs and the two GCMs (ACCESS-ESM1.5 and CESM2) analyzed. For the deeper model rows, we add together the values for DJF ONI and Annual Niño 1+2 to create a single ENSO term. The DJF Atlantic and Indian Ocean contributions are also summed. Percentages indicate the fractional contribution of each driver to the total observed 2024 *F*_P≥99_ anomaly, calculated by scaling the GCM-derived regression coefficients by the observed 2024 predictor values.

Model setup	GCM	GMST (warming)	ENSO (PAC)	Regional (ATL/IND)	Residual
Simple model (two terms)	ACCESS	42.3 ± 7.3%	16.9 ± 7.4%	–	40.8%
	CESM2	38.7 ± 7.8%	27.1 ± 5.6%	–	34.2%
Deeper model (five terms)	ACCESS	36.0%	10.5%	22.8%	30.7%
	CESM2	29.1%	12.7%	45.1%	13.1%

Extending the regression to include additional SST predictors (annual Niño 1+2 and detrended DJF tropical SST anomalies for the Atlantic and Indian Oceans) improves model fit (*R*^2^ = 0.61 to 0.75) and reduces the 2024 residual. The inclusion of these regional predictors redistributes variance previously assigned to GMST and ONI. Because the regional SST anomalies are detrended, this redistribution suggests that the Atlantic and Indian Oceans provide a dynamical boost to extremes that is distinct from their contribution to the global thermodynamic mean. In this expanded framework, ∼29 to 36% of the 2024 *F*_P≥99_ anomaly projects onto GMST. However, the role of tropical SSTs remains highly GCM dependent, with tropical Atlantic variability playing a much larger role in CESM2 (31.6 ± 11.7%) than it does for ACCESS (15.1 ± 12.8%). This divergence likely arises from differences in simulated inter-basin coupling: CESM2 more frequently exhibits co-occurring SST anomalies across basins (Fig. 6) and consequently explains a larger fraction of the 2024 anomaly (∼87%) compared to ACCESS (∼69%).

This predictive gap is mirrored in an observational-only ordinary least squares (OLS) model. We find that a simple model requiring only GMST and Niño 1+2 can explain a large portion of the observed variability (*R*^2^ = 0.58). For 2024, this model predicts an *F*_P≥99_ of 1.14%, ∼75% of which projects onto the GMST term, leaving a residual of ∼0.06%. If the expanded regression model is forced onto the short observational record, the secondary basin coefficients fail to emerge as statistically robust. This reflects observational sampling limitations. Because synchronized multi-basin warmth is rare in the short observational record, regression cannot statistically decouple these individual drivers. Conversely, the thousands of simulated years in the LENSs provide the statistical power to repeatedly sample these rare states and isolate their unique contributions. Together, these results suggest that the 2024 *F*_P≥99_ record was not merely a linear response to warming, but was intensified by a rare synchronization of multi-basin tropical SST anomalies, consistent with additional dynamical amplification.

## DISCUSSION

We find that 2024 saw a record-high frequency of heavy precipitation events, both globally and over land areas (Fig. 1). This continues a long-term upward trend observed in recent decades, roughly equal to 0.03%/decade for events greater than the 99th percentile. Models and observations generally agree on the global trend magnitude, but they diverge in some spatial features. Similar behavior is evident for more extreme (>99.9th percentile) precipitation events, albeit with larger uncertainty.

The extreme conditions in 2024 were largely shaped by the combined influence of long-term global warming, which has elevated water vapor levels in the atmosphere, a strong El Niño, and multi-basin ocean warmth. Throughout the year, these factors played differing roles in controlling heavy precipitation. A strong El Niño, alongside concurrent tropical Atlantic and Indian Ocean warmth, played a pivotal role in elevating *F*_P≥99_ during the first half of 2024. Although these tropical SST anomalies largely decayed by mid-year [except for the Indian tropical basin (IND) anomaly], their influence was prolonged into boreal summer through systematic lags in translating equatorial SST anomalies to global tropospheric temperature and moisture (*37*–*39*). This effect was then enhanced by the long-term warming trend, which exerts a much stronger, dominant influence on precipitation extremes during boreal summer. Consequently, the spatial structure of precipitation extremes in 2024 differed from past strong El Niños. Unlike the EP events of 1983 and 1998, *F*_P≥99_ was muted along the equatorial EP and instead amplified across the high latitudes and the NH tropics. This unique pattern arose because the rare co-occurrence of warm anomalies across additional tropical Ocean basins redistributed tropical convection and altered extratropical teleconnections. Thus, 2024 illustrates how global warming interacts with evolving patterns of tropical SST variability to shape hydroclimate risk.

The GCM LENSs indicate that years such as 2024 are rare but not unprecedented, equal to the 91.4th percentile of all simulated 2024 values across all members. However, simulated analogs typically require stronger El Niño amplitude (2.8K versus 1.9K DJF ONI; Fig. 5), concurrent tropical SST anomalies across multiple basins, or an El Niño that intensifies throughout the year. This suggests that synchronization of tropical SST variability can substantially amplify precipitation extremes beyond what would be expected from ENSO amplitude alone.

If current trends persist, we could expect to see the record-setting *F*_P≥99_ of 2024 become a typical year by mid-century (fig. S12). Furthermore, years with *F*_P≥99_ below the historical baseline average may become virtually absent in the near future. Despite anomalous behavior in 2024, the inclusion of an additional 7 years in the observational record does not substantially alter the constrained estimates of future *F*_P≥99_ change proposed in T22 because the observed trend in *F*_P≥99_ remains similarly placed within GCM spread. A major limitation encountered during past work was a lack of near-real-time (NRT) observational datasets with global coverage. This work manages to exploit recent improvements to the latency of daily precipitation datasets to deliver information about hydrologic cycle change in a timelier manner. Future work should seek to build a framework for improved tracking of global hydrologic cycle change, similar to what exists for temperature where analyses are promptly released by a collection of government agencies and data providers shortly after each year.

## MATERIALS AND METHODS

### Experimental design

The primary objective of this study is to assess observed change in the frequency of precipitation extremes over recent decades and understand the drivers contributing to record setting precipitation during 2024. We also seek to evaluate the conditions required for GCMs to reproduce the observed precipitation statistics. The datasets and methods used are described below.

### CMIP simulations

We use daily precipitation output from LENSs of four Coupled Model Intercomparison Project Phase 6 (CMIP6) GCMs (*51*). This includes the first 50 members of the CESM2 LENS (*52*), the first 25 members of the CanESM5 LENS (*53*), 20 members of the E3SM2 LENS (*54*), and all 40 members of the ACCESS-ESM1.5 LENS (*55*, *56*). In the case of CanESM5, we are unable to retrieve daily precipitation data from simulations 26 to 50. The analysis featured here relies on precipitation time series that are stitched together from the historical (1850 to 2014) and SSP3-7.0 (2015 to 2100) scenarios. However, we primarily use the most recent 47 years to compare with the observational record. It should be noted that the choice of emissions scenario does not have a large impact over 2015 to 2025. LENSs are preferred for this type of analysis because they allow for both better extraction of the forced signal (for trend analysis) and sampling of internal variability (for assessing rare events). Individual GCMs are assessed at their original output resolutions throughout. We also use monthly SST output (variable “tos”) from the models for analysis of ENSO.

### Observations

We primarily rely on daily precipitation from the MSWEP product, version 2.8 (*31*), which has a resolution of 0.1°. The standard MSWEP product provides data from 1979 to 2020, whereas the NRT product extends the record through 2024. The standard version is derived from a combination of satellite (IMERG and GridSat), gauge (GSOD, GHCN-D, and several national databases), and model-based (ERA5) precipitation datasets. The NRT product uses similar satellite (GSMaP and IMERG) and model-based (ERA5 and GDAS) inputs but lacks daily gauge corrections (*57*). Correction factors are applied to the NRT data to ensure consistency with the standard product. Compared to version 2.2 used in a prior work (*25*), version 2.8 includes an extended temporal record, additional data sources, and improved quality control procedures. MSWEP has demonstrated strong performance in multi-dataset evaluations (*58*, *59*). Version 2.2 was previously shown to exhibit a terrestrial *F*_P≥99_ trend that was in line with other datasets (CPC, CHIRPS, and REGEN) (*25*). Although some differences are present in the global mean *F*_P≥99_ time series between versions 2.8 and 2.2, the overall trend remains largely unchanged.

We applied minimal corrections to the dataset where strong evidence of error was present. Specifically, daily precipitation values exceeding 1800 mm were masked, consistent with the upper bounds of historically observed point measurements, which addressed several unrealistically large values on 2 days in 1996. In addition, data from several days in late February 2009 were excluded due to irregular and likely erroneous precipitation patterns. Although such corrections improve data plausibility, we acknowledge that some artefacts may persist. Accordingly, we recommend interpreting regional-scale patterns rather than the change at individual grid cells.

To complement MSWEP, we also use two additional daily land-only precipitation datasets with low latency: the CPC Global Unified Gauge-Based Analysis of Daily Precipitation and the Climate Hazards Group InfraRed Precipitation with Station data (CHIRPS version 2.0). The CPC dataset (*60*, *61*) spans 1979 to present and is entirely gauge based, whereas CHIRPS begins in 1981 and combines satellite and reanalysis data with interpolated 5-day gauge observations (*62*). The spatial resolutions and domains of these datasets differ: CPC is available globally (90°S to 90°N at a 0.5° resolution, whereas CHIRPS covers 50°S to 50°N at a 0.25° resolution. (50°S to 50°N data should serve as a good approximation for global values as the annual mean *F*_P≥99_ for these domains are strongly correlated in MSWEP.) Although the CPC dataset has demonstrated good performance in an intercomparison study (*58*), its exclusive reliance on gauge data can lead to inaccuracies in sparsely monitored regions. On the other hand, CHIRPS may underestimate extremes due to the use of 5-day gauge data (rather than daily), which limits its ability to resolve short duration events.

Although the three datasets overlap beginning in 1981, low inter-dataset agreement in *F*_P≥99_ during 1981 to 1982 led us to exclude these years from trend analysis. Compared to MSWEP, the other datasets (especially CPC) suggest higher *F*_P≥99_ and *F*_P≥99.9_ in the early portion of their records. Much of the anomalous behavior in CPC stems from large interpolation signatures around remote gauges (primarily in Africa and Asia), which tends to artificially inflate the *F*_P≥99_ (even exceeding 2024 values) more than for the other datasets. Agreement among the datasets improves substantially after 1982, so we choose to assess the multi-dataset average over the 1983 to 2024 period in the main text. During this time, all datasets show broadly consistent *F*_P≥99_ trends, with the strongest increase in CPC and the weakest in CHIRPS. This ordering of trends also holds for *F*_P≥99.9_, although inter-dataset spread is larger (ranging from 0.001 to 0.010%/decade). We speculate that the muted trend in CHIRPS may relate back to its use of 5-day gauge data, which leads to overly smooth precipitation.

### ENSO analysis

We primarily characterize the state of ENSO using a proxy for the ONI. Here, this is calculated as the 3-month running mean of detrended SST anomalies over the Niño 3.4 region (5°S to 5°N, 120° to 170°W) (*63*). For the GCMs evaluated here, ONI is computed from monthly SST data spanning 1950 to 2025. Consistent with the timing of typical ENSO peaks, we primarily analyze ONI values averaged over DJF. The observed ONI value for DJF 2024 of 1.9K is taken from the NOAA (*63*), where a maximum value of 2.1K occurred during November-December-January. To compliment the ONI analysis, we also calculate the Niño 1+2 (0° to 10°S, 80° to 90°W) and Niño 4 (5°S to 5°N, 150°W to 160°E) indices, which are the eastern-most and western-most of the ENSO regions. In evaluating the characteristics of the recent El Niño relative to past strong El Niños, we also use SST and SLP data from the ERA5 global reanalysis dataset (*64*). The spatial structure of SSTs is further analyzed through detrended tropical basin mean calculations for the Pacific, Atlantic, and Indian Oceans (region domains shown in Fig. 4).

### Precipitation frequency metrics

We follow the framework developed by ref. (*25*) for measuring changes in the frequency of extreme precipitation. They consider precipitation at or above the 99th percentile as “extreme” because it represents a rare event and allows for reasonable sampling of events per year. Moreover, future changes above this threshold have been shown to be large and consistent in sign across most latitudes. Here, we expand on this approach to also consider a higher threshold for extremity, events at or above the 99.9th percentile. To differentiate these results, we refer to the 99th percentile as heavy precipitation and the 99.9th percentile as extreme precipitation.

To calculate the historical change in *F*_P≥*n*_, we derive global maps of the *n*th percentile of daily precipitation over a fixed historical climatology (1980 to 2014) for each dataset. (We maintain this climatological period so the results can be easily compared to T22.) Daily precipitation spanning 1979 to 2024 is then compared to the percentile maps to locate areas where a heavy, or extreme, event has occurred. These exceedances are recorded as binary values at each grid cell and averaged across time (annually) and area-weighted space to develop a global mean time series of *F*_P≥*n*_. This global metric represents the total space-time averaged fraction of daily exceedances. This process is repeated for each realization of a LENS. In certain figures, the annual means from these multiple realizations are averaged to minimize the influence of natural variability. To complement this global average and evaluate the annual spatial footprint of these events, we also calculate local annual exceedance counts. Assuming independent daily occurrences, local annual counts follow a binomial distribution [*Bin*(365, 0.01) for heavy and *Bin*(365,0.001) for extreme events], yielding precise theoretical baselines for the fraction of locations expected to cross specific thresholds in a single year. To derive trends, we use OLS regression. Trend significance is assessed using a Mann-Kendall test. Last, we use all available days to calculate percentiles. This avoids complications arising from changes in the amount of rainy days in a year (*65*).

As expected for a nonstationary exceedance-based metric, the absolute magnitude of *F*_P≥*n*_ trends depends on the baseline period used to define the *n*th percentile threshold. Across reasonable baseline choices (1980 to 2010, 1980 to 2014, and 1980 to 2024), observed trends in *F*_P≥99_ remain positive (0.025 to 0.033%/decade), and 2024 remains the most extreme year on record (*F*_P≥99_ = 1.17 to 1.21%) (fig. S13). The baseline choice has a greater influence on observed *F*_P≥99.9_ trends, which span 0.0015 to 0.0048%/decade).

### Regression analysis

To estimate how the 2024 *F*_P≥99_ anomaly projects onto long-term warming and ENSO variability, we apply a multiple linear regression model

$$F_{P\geq 99,t}=\beta_{0}+\beta_{1}{\text{GMST}}_{t}+\beta_{2}{\text{ONI}}_{t}+\varepsilon_{t}$$ (1)

where GMST is the annual GMST anomaly, and ONI is the DJF ONI. GMST anomalies are computed relative to a 1980 to 2014 baseline. The model is fit using OLS regression to annual data from the two LENSs that best reproduce the observed *F*_P ≥99_ trend (CESM2 and ACCESS-ESM1.5). To ensure physical consistency, regressions are performed separately for ensemble members whose historical *F*_P≥99_ trends fall within ± 0.005 of the observed value (0.031%/decade). For each qualifying ensemble member, regression coefficients are estimated using data from 1979 to 2025. The 2024 projected contributions are obtained by multiplying the fitted coefficients by the observed 2024 anomalies (GMST = 0.845K; ONI = 1.9K), with the observed GMST anomaly derived from GISTEMPv4 (*66*, *67*). Individual contributions are expressed as a percentage of the total observed 2024 *F*_P≥99_ anomaly (0.2% above the 1980 to 2014 mean). Reported contribution ranges reflect the distribution across qualifying ensemble members.

To evaluate whether additional basin-scale variability contributes to the 2024 extremity, we repeat the regression with an expanded predictor set that includes annual Niño 1+2, detrended DJF tropical SST anomalies for the Atlantic and Indian Oceans (as displayed in Fig. 4 from 20°S to 20°N). This deeper model is used to assess sensitivity of the partitioning to predictor selection and to diagnose the role of multi-basin SST variability. This model also relies only on the members that resemble the observed historical *F*_P≥99_ trend.
