# Supplementary material for: Record-setting heavy precipitation occurrence in 2024
Source: Sci Adv. 2026 Oct 9;12(41):eaeb1692. doi: 10.1126/sciadv.aeb1692 (PMC13651887; doi:10.1126/sciadv.aeb1692)
Supplement: Supplementary file 1 [file sciadv.aeb1692_sm.pdf]

Science Advances
AAAS

Supplementary Materials for

Record-setting heavy precipitation occurrence in 2024

Chad W. Thackeray *et al.*

Corresponding author: Chad W. Thackeray, cwthackeray@ucla.edu

Sci. Adv. **12**, eaeb1692 (2026)
DOI: 10.1126/sciadv.aeb1692

This PDF file includes:

Figs. S1 to S13

Supplemental Figures

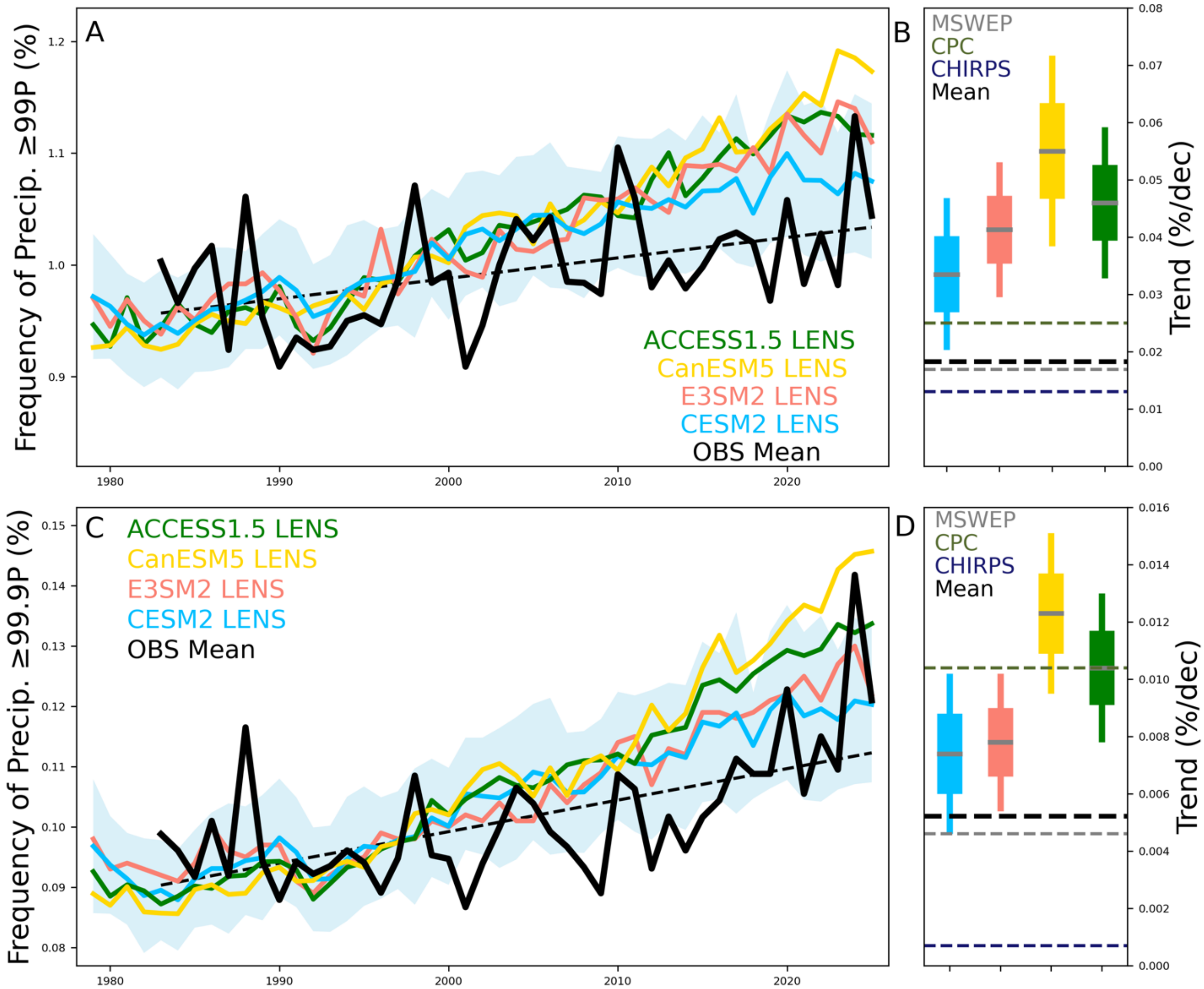

Fig. S1. Historical time series of terrestrial (land only) heavy precipitation frequency from observations and models. Comparison of the observed (black line) and simulated (colored lines) frequency of precipitation events greater than or equal to the (**A**) 99th percentile, (**C**) 99.9th percentile. As in Figure 1, panels **B** and **D** show the distribution of historical trends for each dataset. Various dashed lines in panels **B** and **D** represent different observational datasets (MSWEP, CPC, CHIRPS) while the black dashed line represents the mean trend. Note that we limit the observational record to start in 1983 because of large inter-dataset uncertainty before this point (particularly for $F_{P\geq99.9}$: see Methods).

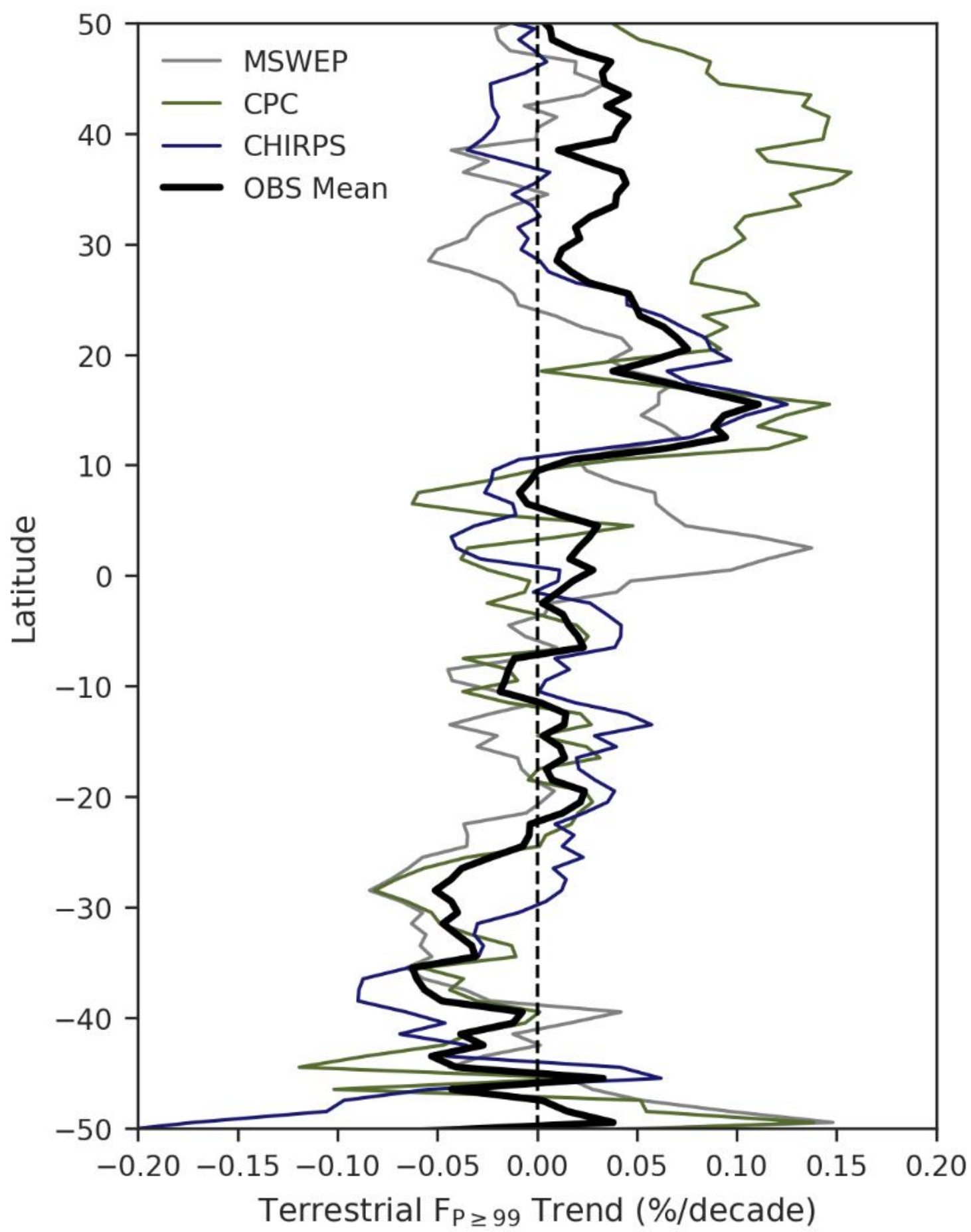

Fig. S2. Zonal mean terrestrial $F_{P\geq99}$ trends. Similar to Figure 2B, but for the various observational datasets across their common domain (50S-50N). Trends are computed for the 1983-2025 period (see Methods).

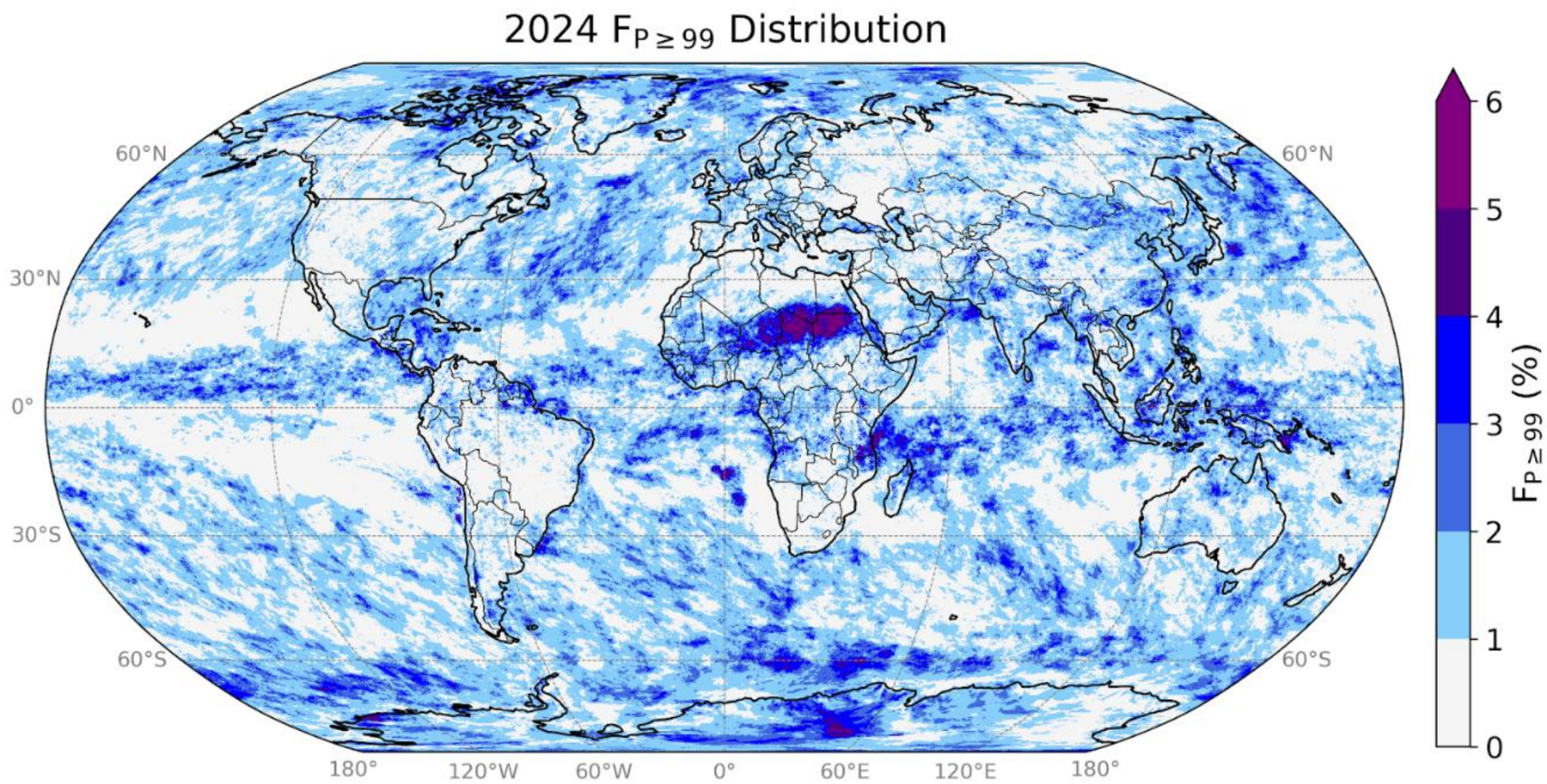

Fig. S3. Map of observed $F_{P\geq99}$ from 2024. Similar to Figure 3A, but for heavy precipitation, and the color bar is striated to illustrate regions where less or more heavy precipitation events occurred than expected (~1% in any given baseline year).

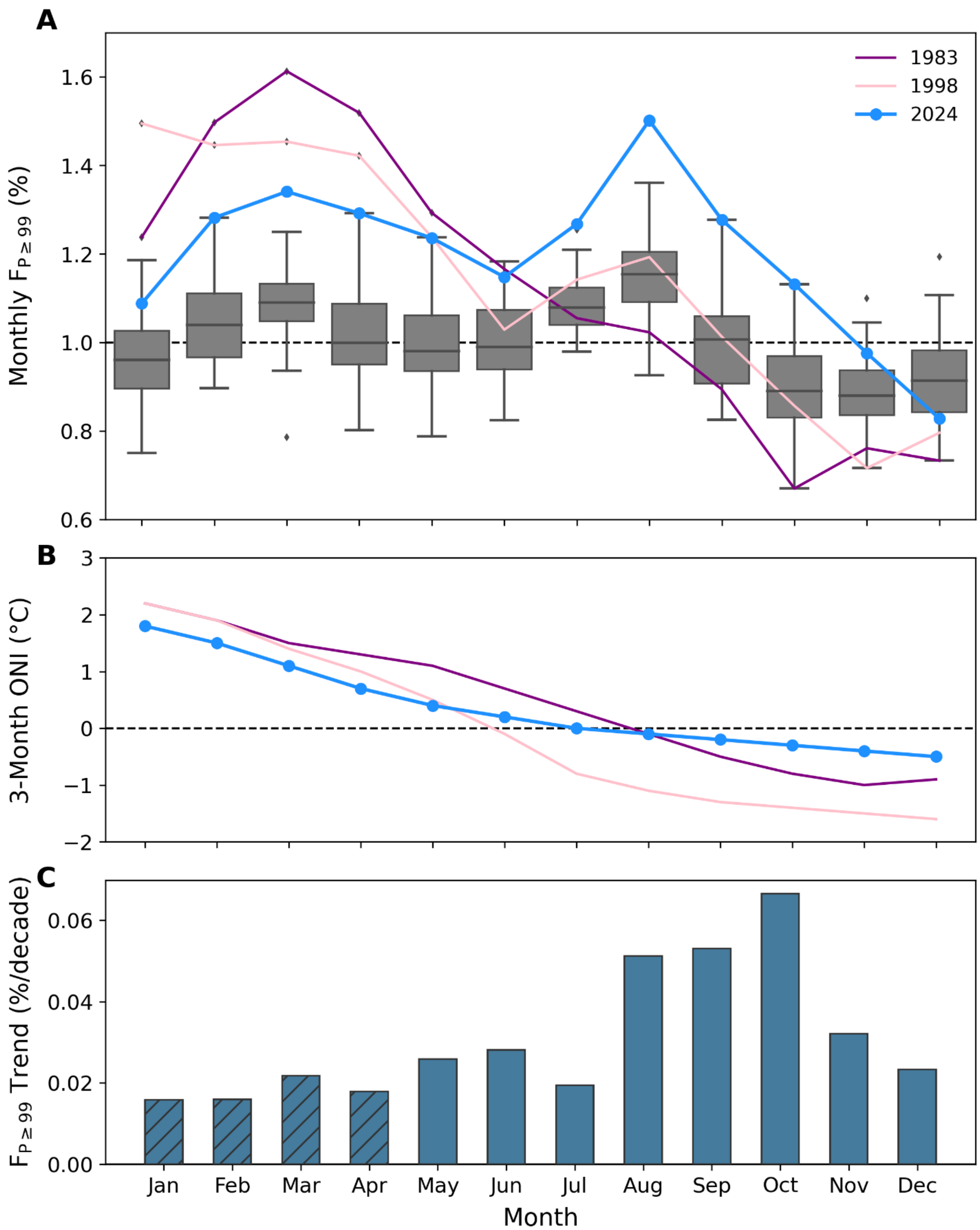

Fig. S4. Seasonal breakdown of historical global mean $F_{P\geq99}$ from MSWEP. (**A**) Boxplot of monthly mean $F_{P\geq99}$ over the 1979-2025 period. The 1983, 1998, and 2024 $F_{P\geq99}$ and (**B**) 3-month mean Oceanic Niño Index are also illustrated by the colored lines. Data points deemed outliers are shown by grey diamonds. Historical monthly trends in $F_{P\geq99}$ are shown in panel (**C**) (derived for 1979-2025) with statistical insignificance shown by hatching. The strongest trends illustrate the time of year when the long-term warming trend is having the biggest impact, while the start of the year is heavily influenced by ENSO.

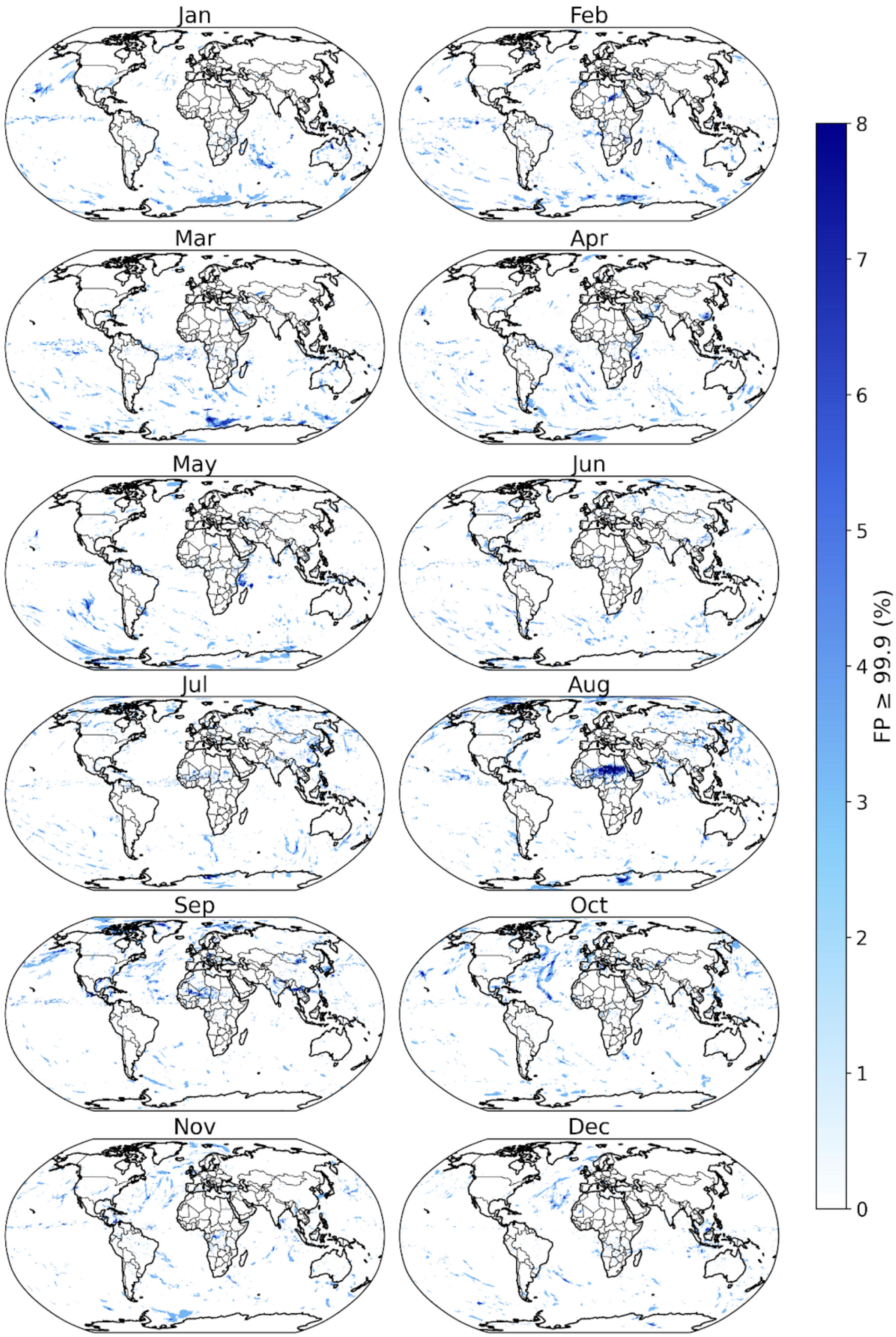

Fig. S5. Maps of monthly mean $F_{P\geq 99.9}$ for 2024 according to MSWEP. Similar to Figure 3, but the time averaging is done on a monthly timescale instead of annual. Thus, this shows the percentage of days in the month with precipitation of at least the 99.9th percentile.

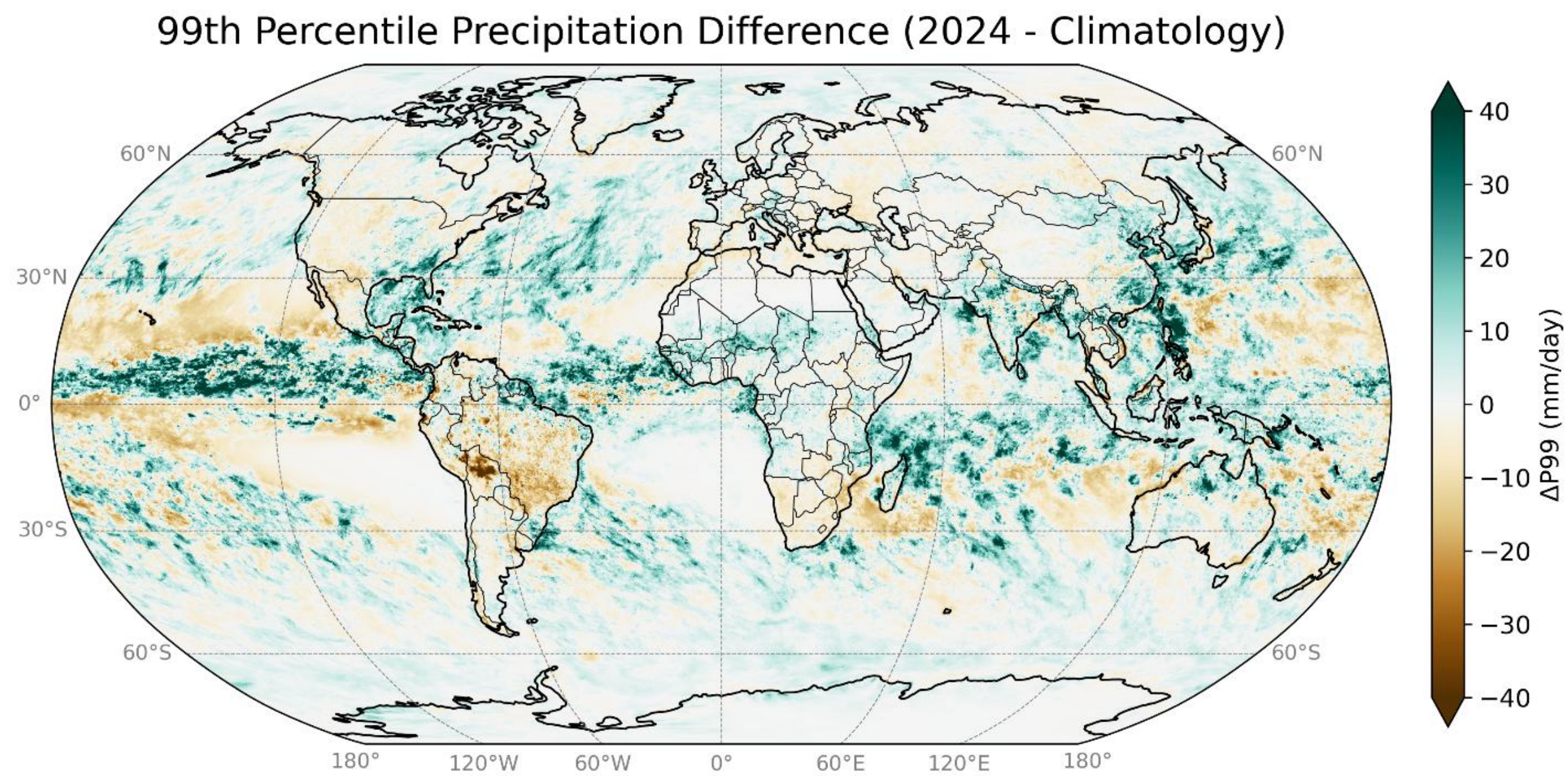

Fig. S6. Observed heavy precipitation anomaly in 2024. Map of the difference in heavy precipitation (99th percentile) magnitude (mm/day) in 2024 relative to the historical climatology (1980-2014).

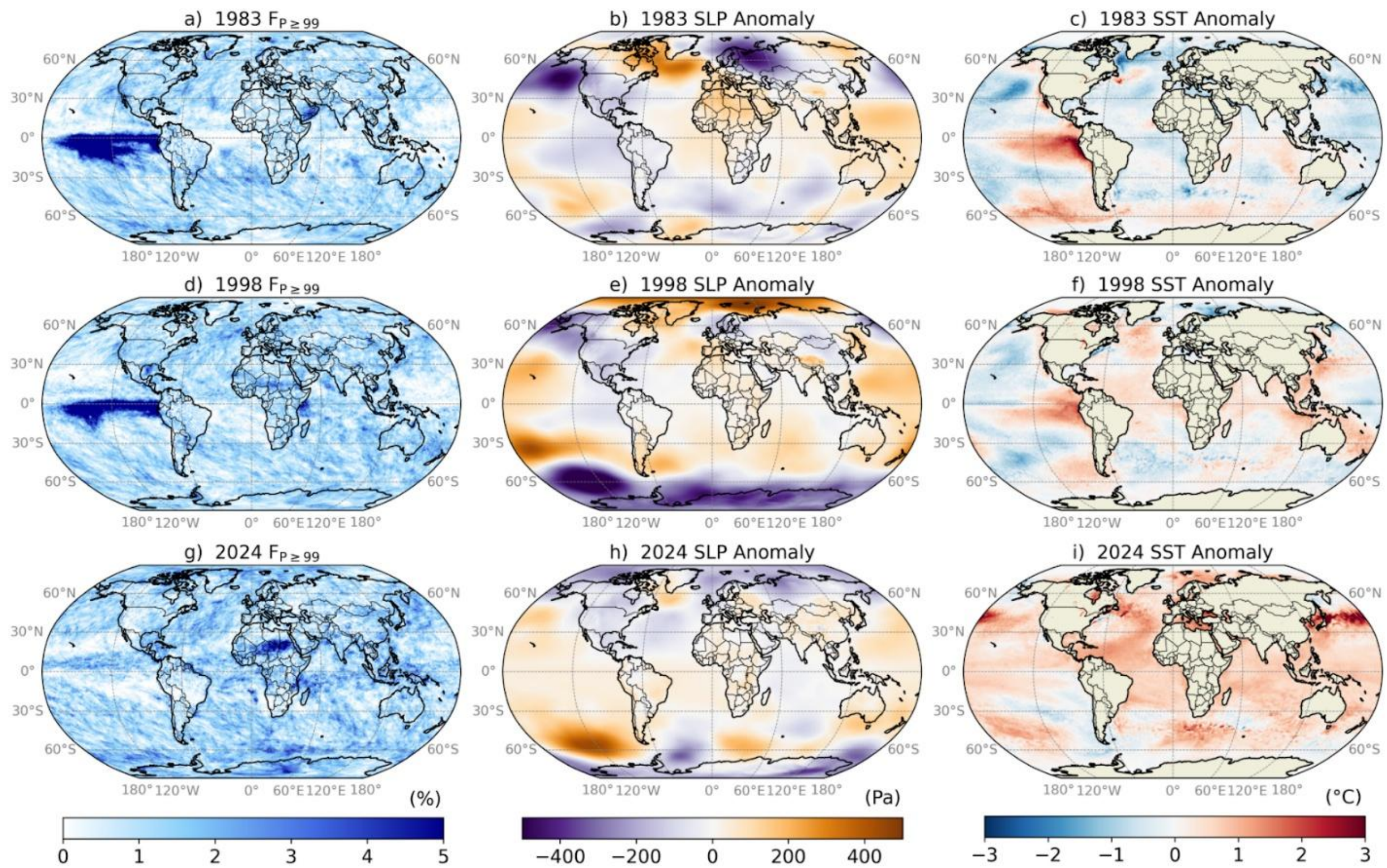

Fig. S7. Conditions during select strong El Niño years. Maps of annual mean (**A, D, G**) $F_{P\geq99}$, (**B, E, H**) sea level pressure anomaly, and (**C, F, I**) sea-surface temperature anomaly from 1983, 1998, and 2024. Anomaly fields are calculated relative to the 1979-2024 climatology. These years were selected for their anomalous $F_{P\geq99}$.

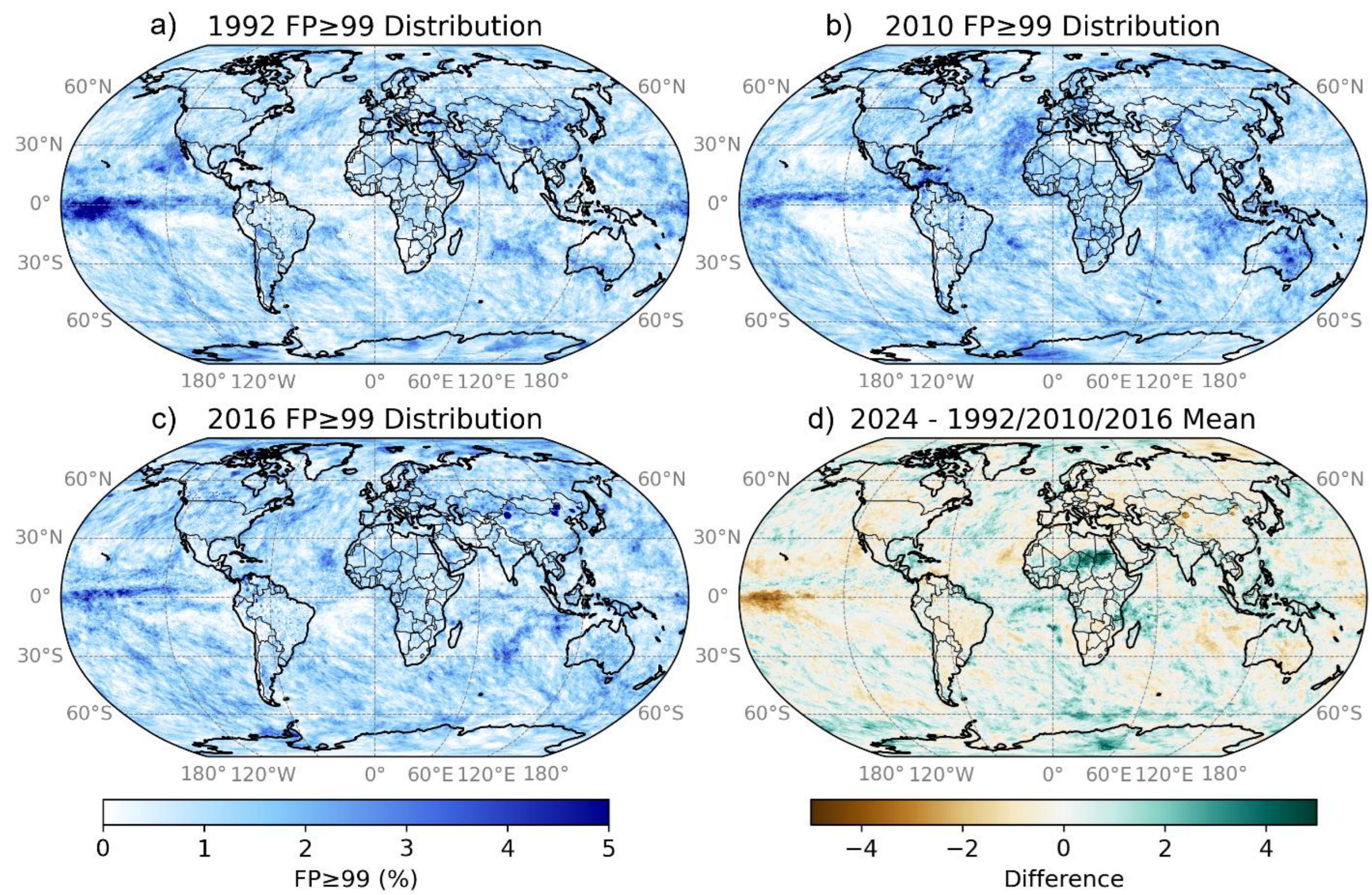

Fig. S8. Comparison with $F_{P\geq99}$ during Central Pacific El Niño events. (**A-C**) Spatial distribution of $F_{P\geq99}$ for a collection of El Niño events with Central Pacific tendencies. (**D**) difference between $F_{P\geq99}$ in 2024 and the mean of the three years shown (1992, 2010, 2016).

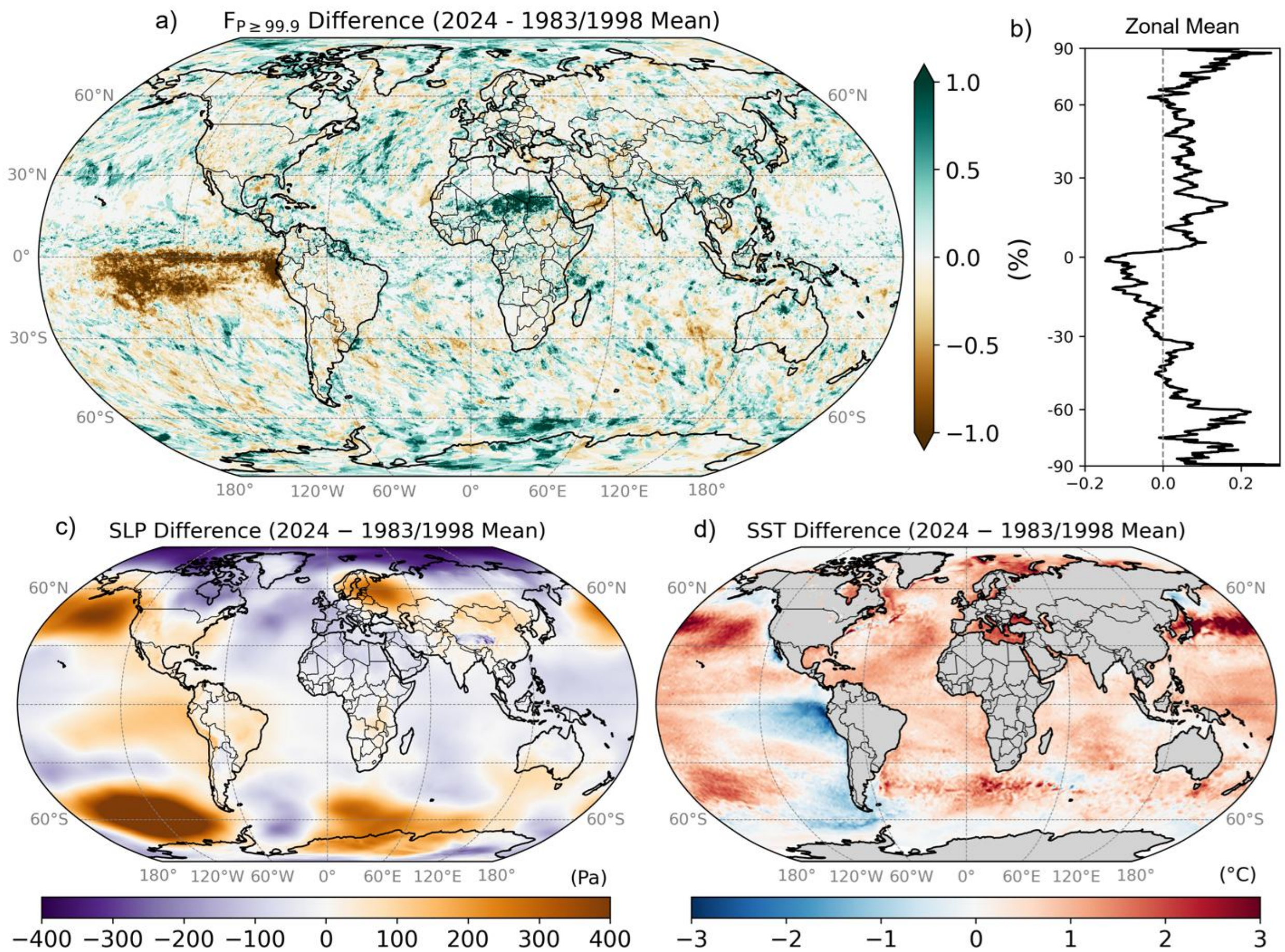

Fig. S9. Comparison of conditions in 2024 to prior strong El Niño years. (**A**) Map of the difference in $F_{P\geq99.9}$ between 2024 and the average of 1983 and 1998. (**B**) Line plot showing the zonal average at each latitude. Maps of the difference in annual-mean (**C**) sea-level pressure and (**D**) sea-surface temperature. These years were selected for their anomalous behavior in Figure 1.

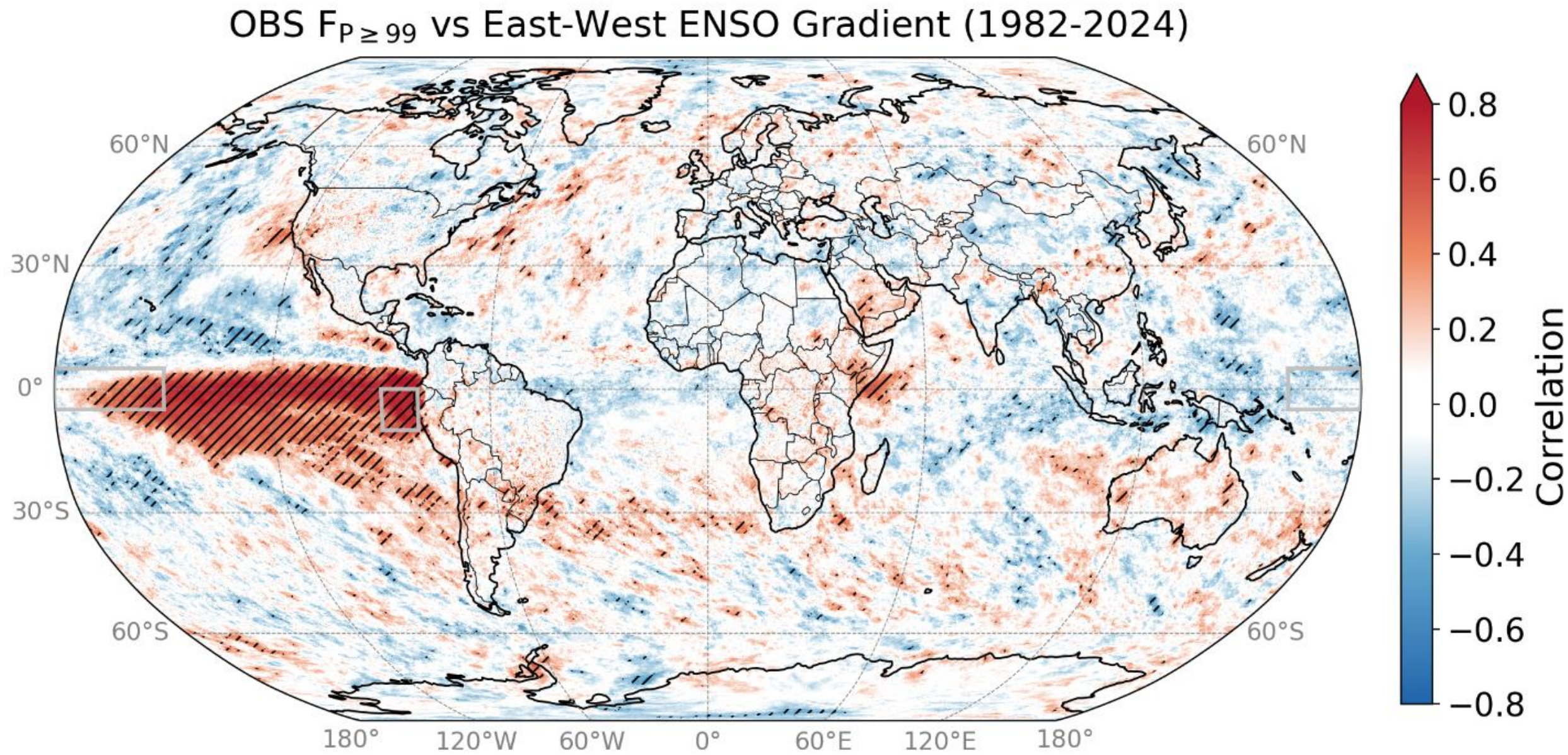

Fig. S10. Correlation between local $F_{P\geq99}$ and the ENSO gradient. Temporal correlation between observed local $F_{P\geq99}$ and the East to West gradient in ENSO for the years 1982-2024. The ENSO gradient is calculated as the annual mean Niño1+2 minus Niño4. The two Niño regions are shown by grey boxes. Stippling shows areas of significance ($p<0.05$). Similar patterns exist when isolating for only El Niño or La Niña years.

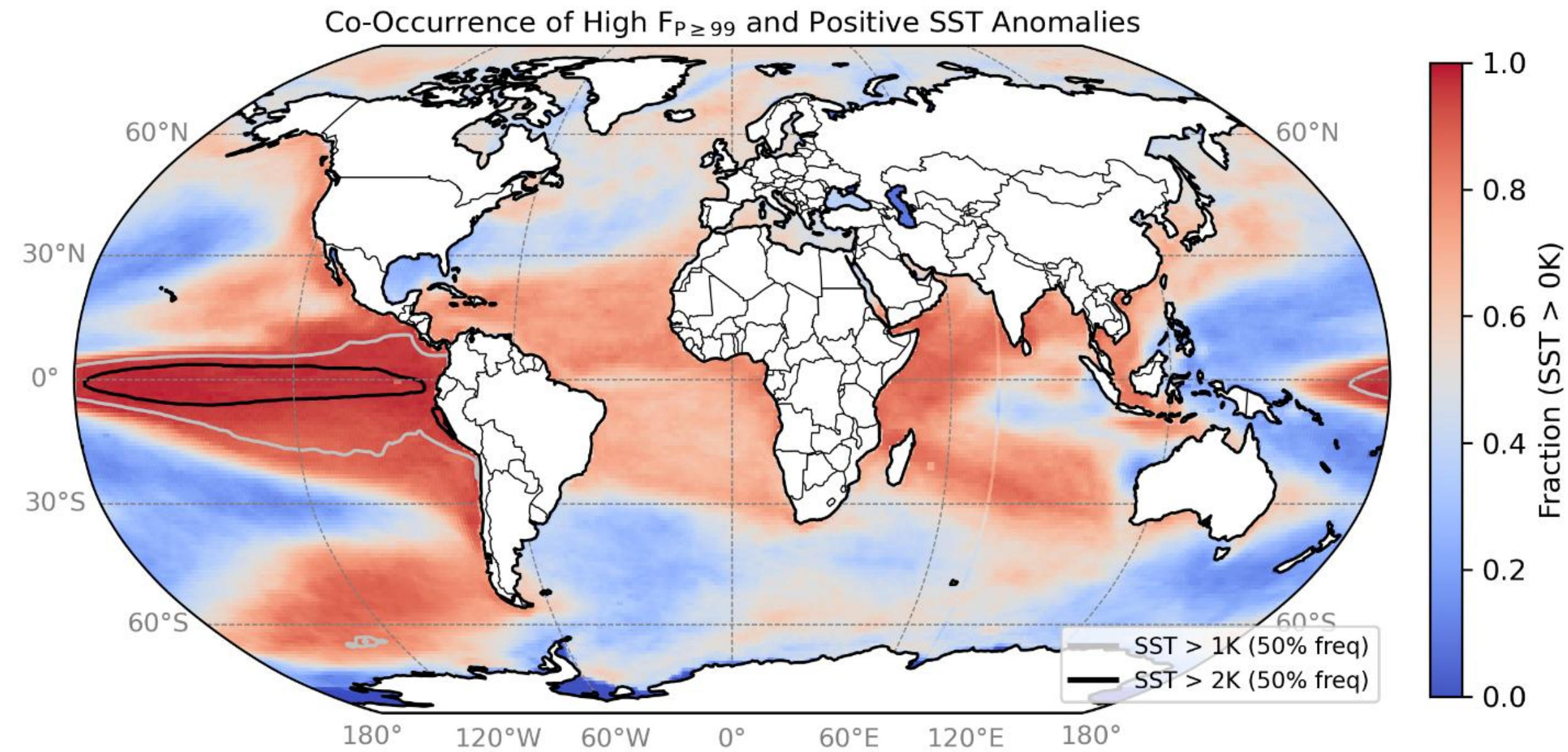

Fig. S11. Co-occurrence of high $F_{P\geq99}$ and positive SST anomalies. Map of the fraction of simulated high $F_{P\geq99}$ years from the historical period that feature positive detrended DJF SST anomalies. Contours illustrate areas where at least half of these simulated years have an SST anomaly above 1K (grey line) or 2K (black line) The high $F_{P\geq99}$ years are a collection of 125 simulated years where the $F_{P\geq99}$ matches or exceeds the 2024 observed value.

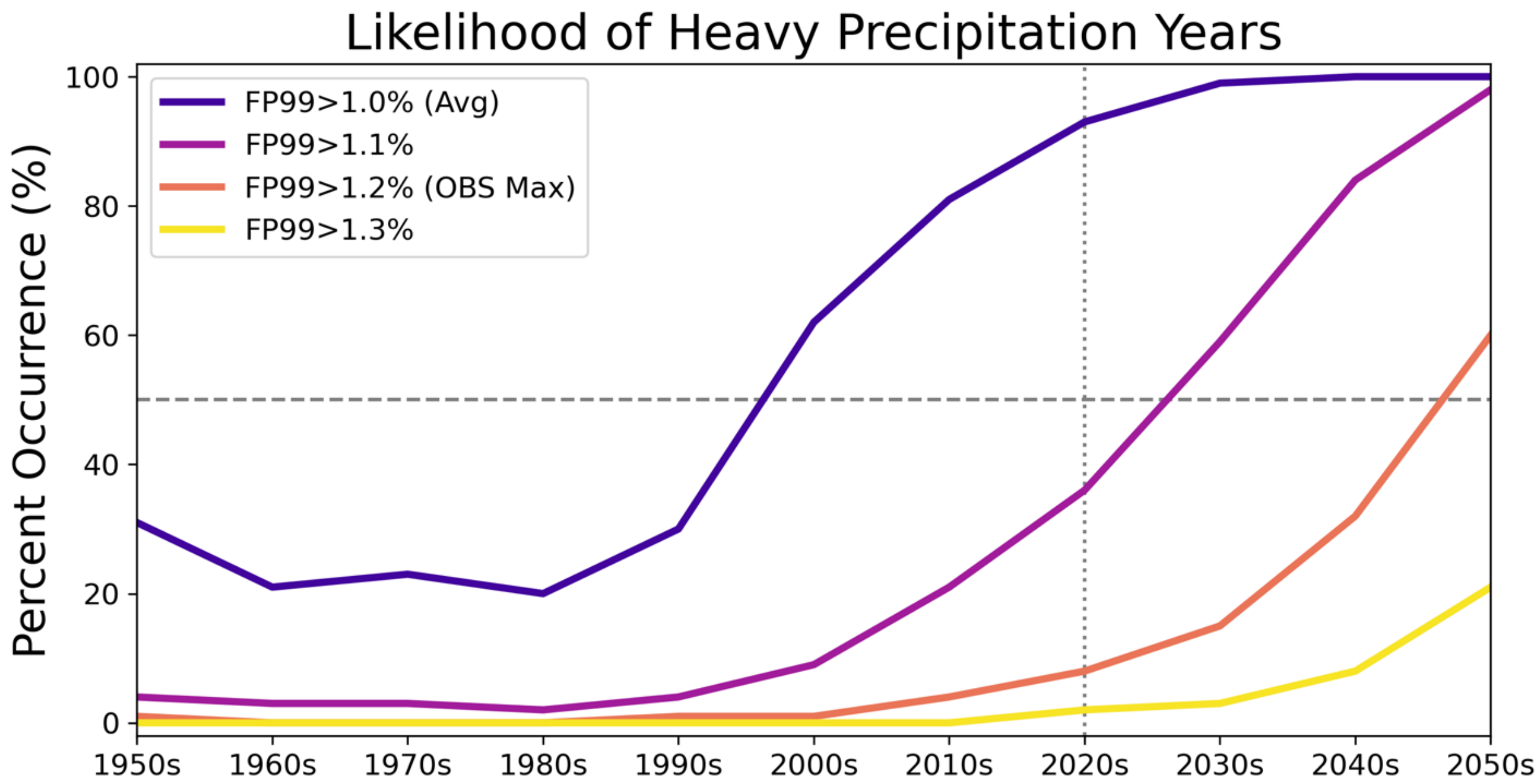

Fig. S12. Historical and near-future likelihood of a simulated year with varying levels of $F_{P\geq99}$. Percent occurrence of simulated years per decade above various $F_{P\geq99}$ thresholds from the CESM2 and ACCESS-ESM1.5 large ensembles. We take the average of the likelihood from the two large ensembles that best capture the observed trend in $F_{P\geq99}$. Colored lines illustrate differing thresholds of $F_{P\geq99}$ including that of an average year over the baseline climate (1.0%) and the observed maximum from 2024 (1.2%). This metric is calculated for each year of the time series and then averaged across the decade to reduce noise.

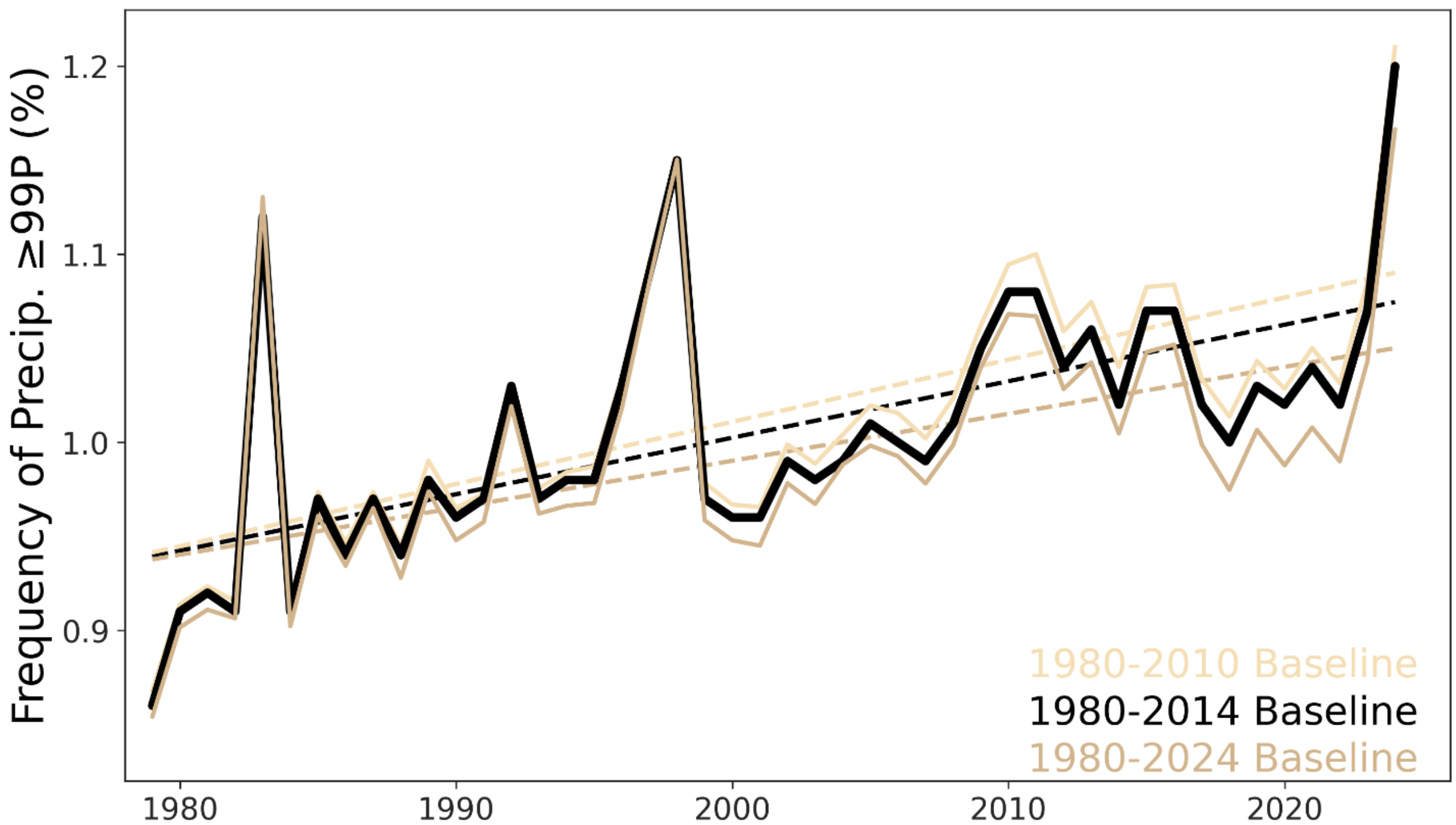

Fig. S13. Sensitivity of observed $F_{P\geq99}$ from 1979-2024 to different historical baseline periods. Time series of annual mean $F_{P\geq99}$ from MSWEP calculated using various reasonable historical baselines (1980-2010, 1980-2014, and 1980-2024) to determine the 99th percentile thresholds.
